# Hsa_circ_001680 affects the proliferation and migration of CRC and mediates its chemoresistance by regulating BMI1 through miR-340

**DOI:** 10.1186/s12943-020-1134-8

**Published:** 2020-01-31

**Authors:** Xiangyu Jian, Han He, Jiehong Zhu, Qi Zhang, Zhongxin Zheng, Xiangjing Liang, Liuyan Chen, Meiling Yang, Kaiyue Peng, Zhaowen Zhang, Tengfei Liu, Yaping Ye, Hongli Jiao, Shuyang Wang, Weijie Zhou, Yanqing Ding, Tingting Li

**Affiliations:** 1 Department of Pathology, Nanfang Hospital, Southern Medical University, Guangzhou, 510515 Guangdong China; 2 Department of Pathology, School of Basic Medical Sciences, Southern Medical University, Guangzhou, Guangdong China; 3 Department of Hematology, Nanfang Hospital, Southern Medical University, Guangzhou, Guangdong China

**Keywords:** Has-circ_001680, miR-340, Irinotecan, BMI1, Stem cell, Chemotherapy resistance

## Abstract

**Background:**

Accumulating evidence indicates that circular RNAs (circRNAs) act as microRNA (miRNA) sponges to directly inhibit specific miRNAs and alter their ability to regulate gene expression at the post-transcriptional level; this mechanism is believed to occur in various cancers. However, the expression level, precise function and mechanism of circ_001680 in colorectal carcinoma (CRC) are largely unknown.

**Methods:**

qRT-PCR was used to detect the expression of circ_001680 and miR-340 in human CRC tissues and their matched normal tissues. Bioinformatics analyses and dual-fluorescence reporter assays were used to evaluate whether circ_001680 could bind to miR-340. Circ_001680 overexpression and knockdown cell lines were constructed to investigate the proliferation and migration abilities in vivo and in vitro through function-based experiments, including CCK8, plate clone formation, transwell, and wounding healing assays. The relationships among circ_001680, miR-340 and BMI1 were investigated by bioinformatics analyses, dual-fluorescence reporter system, FISH, RIP and RNA pull down assays. Sphere forming assays and flow cytometry analyses were used to assess the effect of circ_001680 on the stemness characteristics of CRC cells.

**Results:**

Circ_001680 was more highly expressed in of CRC tissue than in matched adjacent normal tissues from the same patients. Circ_001680 was observed to enhance the proliferation and migration capacity of CRC cells. Furthermore, dual-fluorescence reporter assays confirmed that circ_001680 affects the expression of BMI1 by targeting miR-340. More importantly, we also found that circ_001680 could promote the cancer stem cell (CSC) population in CRC and induce irinotecan therapeutic resistance by regulating the miR-340 target gene BMI1.

**Conclusions:**

Our results demonstrated that circ_001680 is a part of a novel strategy to induce chemotherapy resistance in CRC through BMI1 upregulation. Moreover, circ_001680 may be a promising diagnostic and prognostic marker to determine the success of irinotecan-based chemotherapy.

## Introduction

Colorectal cancer (CRC) is one of the most common malignant neoplasms worldwide; the number of CRC cases increases every year, and CRC poses a serious threat to human life and health [1]. The unknown etiology, lack of obvious symptoms in the early stages, and high level of metastasis are important factors leading to the dismal prognosis and high mortality for CRC patients [2]. Although progress has been made in diagnostic and therapeutic strategies, the clinical outcomes and prognoses of CRC patients with advanced-stage disease remain poor [3]. Thus, further research on the molecular mechanisms that regulate the progression and migration of CRC represents a crucial step in the exploration of novel molecular targets, which may help to generate more effective therapies.

Circular RNAs (circRNAs) are a class of RNA molecules that form single-stranded closed loop structures through covalent bonds without 5′ or 3′ free ends [4]. Following the advent of high-throughput sequencing and computational approaches, thousands of circRNAs have been identified. The majority of circRNAs are exonic circRNAs, which are derived from exonic regions of known protein-coding genes by back-splicing [5]. In recent years, an increasing number of studies have identified circRNAs that play crucial roles in tumor carcinogenesis by sponging microRNA (miRNAs) [6]. For example, the well-known circRNA ciRS7 abrogates the tumor suppressive effect of miR-7 to promote the progression of esophageal squamous cell carcinoma [7] and colorectal cancers [8]. Circ_0039569 promotes renal cell carcinoma growth and metastasis by regulating miR-34a-5p/CCL22 [9]. Circ-ZEB1.33 promotes the proliferation of human hepatocellular carcinoma (HCC) by sponging miR-200a-3p and upregulating the expression of CDK6 [10].

MiR-340 has been reported as a tumor suppressor gene that can regulate the cell cycle and affect tumor migration and metastasis in several types of neoplasms, including glioblastoma multiforme [11, 12], non-small cell lung cancer [13], breast cancer [14], ovarian cancer [15] and gastric cancer [16]. Furthermore, the expression of miR-340 in bone marrow negatively correlates with liver metastasis of CRC [17]. Zhang et al. showed that mir-340 suppresses the growth and enhances the chemosensitivity of CRC by targeting RLIP76 [18]. Sun et al. demonstrated that miR-340 inhibits the growth of CRC by neutralizing the Warburg effect by regulating the alternative splicing of the PKM gene [19]. We used a bioinformatics website (http://starbase.sysu.edu.cn/index.php) to predict that miR-340 has binding sequence within many circRNAs. We detected the expression of these circRNAs in miR-340 overexpressed cells and 20 fresh colon cancer tissues. The results showed that only hsa_circ_001680 had a negative correlation with miR-340. Therefore, we suspected potential interactions exist between circ_001680 and miR-340 and we focused our research on circ_001680.

Circ_001680 (circBase ID: hsa_circ_0000598), is a circRNA located at position of chr15:45009906–45,009,989. The gene symbol is B2M. It has not been reported in any tumors. The mechanism of miR-340 and circ_001680 in the progression of CRC has not been elucidated, and the specific function of circ_001680 in the development of CRC requires further study.

In this study, we demonstrated that circ_001680 could promote the proliferation and migration of CRC cells. Furthermore, circ_001680 was shown to inhibit the expression of miR-340 by acting as an RNA sponge. In addition, circ_001680 could upregulate the miR-340 target gene BMI1, promote the cancer stem cell (CSC) population of CRC cells and induce irinotecan chemotherapy resistance. Our results highlight a new molecular mechanism underlying the tumorigenicity of colon cancer cells and suggest that circ_001680 is a potential chemotherapy resistance marker in CRC.

## Materials and methods

### Tissue specimens and cell cultures

Forty-two pairs of freshly CRC specimens and their matched adjacent paracancerous normal colorectal tissues were recruited from the Department of General Surgery, Nanfang Hospital for histological analysis. All tissues were stored in liquid nitrogen for further use. The medical records of the patients were collected, and the following information was obtained: age, sex, pathological stage, T stage, lymph node metastases and distant metastasis.

Human colorectal cell lines (FHC, HCT116, SW480, HCT15, SW620, CACO2, DLD1, LOVO, HT29, HCT8 and RKO) were purchased from American Type Culture Collection Cell Biology Collection and were maintained in the Department of Pathology, Southern Medical University. Cells were cultured in RPMI 1640 (Invitrogen, Carlsbad, CA, USA) or DMEM (Invitrogen, Carlsbad, CA, USA) supplemented with 10% FBS (Invitrogen, Carlsbad, CA, USA) and 1% penicillin/streptomycin (Invitrogen, Carlsbad, CA, USA) at 37 °C with 5% CO_2_.

### Wound healing assay

A total of 5 × 10^5^ cells/well in the logarithmic growth phase were seeded into 6-well plates. When the cell density reached 80 to 90%, a scratch was made in the monolayer in the middle of the well with a 100 μl pipette tip. The tip was kept perpendicular to the bottom of the well to obtain a straight gap. The detached cells were washed away and removed three times a day. Wound healing within the same scraped line was then observed and photographed at the indicated time points (0 h, 24 h, 48 h, and 96 h). Each experiment was repeated three times.

### RNA extraction and qRT-PCR

Total tissue mRNA was extracted from tissues with a mirVana miRNA Isolation Kit (Ambion, Austin, TX, USA) according to the manufacturer’s protocol. Then, we synthesized cDNA from total RNA using the TaqMan miRNA Reverse Transcription Kit (Applied Biosystems, Foster City, CA, USA). qRT-PCR was performed on the Applied Biosystems 7500 Sequence Detection system with IQTM SYBR Green Supermix (Bio-Rad Laboratories, Hercules, CA, USA) and 5 ng of cDNA and 10 pM of each primer. The cycling conditions were set as previously described [20]. The data were normalized to the geometric mean of the housekeeping gene GAPDH or to U6 small nuclear RNA expression and calculated according to the 2^-ΔΔCT^ method. The forward and reverse primer sequences are shown in Additional file 1: Table S1.

### Migration assay

A total of 1 × 10^4^ cells were seeded into the upper Boyden chamber with an 8 μm pore size filter membrane and culture medium supplemented with 10% fetal bovine serum was added to the lower chamber as a chemoattractant. Twenty-four hours later, the cells on the upper filter were gently removed with a cotton swab. The cells that had migrated to the lower surface of the filter were fixed in 4% paraformaldehyde and stained with hematoxylin for 10 min. Then, the cells were washed three times to remove the hematoxylin. The filter membrane was dried with a blower, and the migrated cells (10 random 200× fields per well) were counted. Three independent experiments were performed and the data are presented as the mean ± s.e.m.

### Colony formation assay

Detection CRC cells were digested and seeded directly in 6-well plates (5 × 10^2^ cells/well) for the colony formation assay and cultured in the presence of 10% FBS and 1% penicillin/streptomycin at 37 °C with 5% CO_2_. Two weeks later, the medium was removed, and the plates were washed with phosphate-buffered saline (PBS) three times. The cells were fixed with anhydrous ethanol for 30 min and then stained with hematoxylin for 20 min. The plates were dried with a blower to ensure that high-quality images were obtained. The colonies were defined as > 50 cells/colony.

### Xenograft model in nude mice

For tumourigenesis assays, 2 × 10^6^ cells per mouse were subcutaneously injected into the right dorsal flanks of female BALB/c athymic nude mice (4–6 weeks of age, 18–20 g), which were obtained from the Animal Center of Southern Medical University, Guangzhou, China). The mice were sacrificed at approximately 7 to 8 weeks. The tumors were excised and placed in 10% neutral buffered formalin for 24 h. The tumors were excised and placed in 10% neutral buffered formalin for 24 h [21]. All mice were raised under specific pathogen-free conditions, and all experiments were approved by the Use Committee for Animal Care and were performed in accordance with institutional guidelines. The tumor size was measured using a slide caliper, and the tumor volume was determined by the following formula: 0.44 × A × B^2^, where A represents the diameter of the base of the tumor and B represents the corresponding perpendicular value.

### Immunohistochemistry

The tissues were cut into 4 μm-thick sections, baked at 65 °C for 30 min, and then dewaxed by dimethylbenzene and alcohol. The sections were then deparaffinized with xylene and treated with 3% hydrogen peroxide to attenuate endogenous peroxidase activity. Next, the sections were submerged in citrate buffer for antigen retrieval and incubated with 1% bovine serum albumin (BSA) to block nonspecific binding. Primary antibodies against Ki67 (1:500; ZSGB-BIO, Beijing, China), caspase-3 (1:500; ZSGB-BIO, Beijing, China), CD133 (1:200; Cell Signaling Technology, Danvers, MA, USA), BMI1 (1:200; Cell Signaling Technology, Danvers, MA, USA), and SOX-2 (1:200; Cell Signaling Technology, Danvers, MA, USA) and an appropriate secondary antibody (1:500, ZSGB-BIO, Beijing, China) were used according to the manufacturer’s instructions. The sections were incubated with DAB and hematoxylin and then scored independently by two observers. The score was based on both the proportion of positively stained tumor cells and the intensity of staining.

### Luciferase assays

CRC cells were seeded in triplicate into 24-well plates (1 × 10^5^ cells per well) and then cultured for 24 h. The constructed pGL3-basic luciferase reporter plasmid (1.5 μg, Promega) or the control luciferase plasmid (1.5 μg, Promega) was cotransfected into the cells with the pRL-SV40 plasmid (0.15 μg, Promega) using Lipofectamine 2000 Reagent (Invitrogen). Luciferase and Renilla activities were detected 36 h after transfection using the Dual-Luciferase Reporter Assay Kit (Promega) according to the manufacturer’s protocol. All experiments were conducted at least three times, and the data are presented as the mean ± SD.

### Flow cytometry assay

The indicated CRC cells were incubated with 5 μg/ml irinotecan (Selleck, S2217) or DMSO for 36 h and then placed in a 1.5 ml tube. The cells were then washed twice with PBS and centrifuged at 400 x g for 2 min. The supernatant was discarded, and the cells were resuspended in 60 μl of surface staining buffer (PBS, pH 7.4, 0.1% BSA) containing antibodies against CD44 and CD133 at 1 μg/ml (BD Pharmingen, Franklin Lakes, NJ, USA) and incubated for 30 min at 4 °C. Then, the cells were resuspended in PBS without washing and analyzed on a FACS flow cytometer according to the manufacturer’s instructions. The results were analyzed by FlowJo software.

### Western blot analysis

Western blotting was performed according to a previous study [22]. Protein lysates were prepared, subjected to SDS-PAGE, transferred onto PVDF membranes and blotted according to standard methods using anti-BMI1 (Cell Signaling Technology, Danvers, MA, USA), anti-CD44 (BD Pharmingen, Franklin Lakes, NJ, USA), anti-CD133 (BD Pharmingen, Franklin Lakes, NJ, USA), and anti-SOX2 (Cell Signaling Technology, Danvers, MA, USA); an anti-α-tubulin monoclonal antibody (Sigma, St Louis, MO, USA) served as a loading control.

### Fluorescence in situ hybridization (FISH)

The digoxin-labeled probes specific to circ_001680 and biotin-labeled probes against miR-340 were prepared by Geneseed Biotech, and the sequences are showen in Additional file 1 Table S1. SW480 and HCT116 cells were cultured on coverslips and fixed with 4% paraformaldehyde in PBS for 15 min. The probes were diluted in hybridization solution (Geneseed Biotech, Guangzhou, China) in PCR tubes and were heated at 95 °C for 2 min in a PCR block to denature the probe. The probe was immediately chilled on ice to prevent reannealing. The hybridization solution was drained, and 100 μL of diluted probe per section was added to cover the entire sample. The samples were covered with a coverslip to prevent evaporation and were incubated in the humidified hybridization chamber at 65 °C overnight. The signals were detected by Cy3-conjugated anti-digoxin and FITC-conjugated anti-biotin antibodies (Jackson ImmunoResearch Inc., West Grove, PA, USA). Cell nuclei were counterstained with 4,6-diamidino-2-phenylindole (DAPI). Finally, the images were obtained on a Zeiss LSM 700 confocal microscope (Carl Zeiss, Oberkochen, Germany) [23–26].

### RNA pull-down assays

RNA pull-down assays were performed with PierceTM Magnetic RNA-Protein Pull-Down Kit (Millipore, Billerica, MA, USA) following the manufacturer’s suggestions, biotinylated circ_001680, biotinylated BMI1 (Geneseed, Guangzhou, China, the sequence is shown in Additional file 1: Table S1) or biotinylated negative control (NC) was incubated with the RIP lysates from SW480 and HCT116 cells for 2 h at 25 °C. The circ_001680/miR-340 or BMI1/miR-340 complexes were captured with Streptavidin-coupled Dynabeads for 1 h at 25 °C. Then the complexes were incubated with wash buffer containing proteinase K for 1 h at 25 °C. The complexes in the pull-down were determined using qRT-PCR analysis. All tests were carried out in triplicate [23–26].

### RNA immunoprecipitation

RNA immunopreciptiation (RIP) assay was conducted by an EZ-Magna RIP Kit (Millipore, Billerica, MA, USA) according to the manufacturer’s protocol. The AGO2-RIP experiments were performed in SW480 and HCT116 cells transiently overexpressing miR-340 or miR-NC. Forty-eight hours later, Approximately 1 × 10^7^ cells were collected and dissolved in 100% RIP Lysis Buffer with proteinase and RNase inhibitors, and the RIP lysates were incubated with RIP buffer containing magnetic beads conjugated with human anti-Ago2 antibody or nonspecific mouse IgG antibody (Cell Signaling Technology, USA). Twenty-four hours later, the RNA/bead complex was washed five times and resuspended in buffer supplemented with RNase-free DNase and proteinase K. The immunoprecipitated RNAs were subjected to qRT-PCR to detect the enrichment [23–26]. All tests were carried out in triplicate.

### Tumor sphere formation assays

A mixture culture medium was prepared and included serum-free 1640 medium (Invitrogen), 2% B-27 Supplement (Invitrogen, Carlsbad, CA, USA), 20 ng/ml basal fibroblast growth factor (bFGF) (PeproTech, Rocky Hill, NJ, USA), 20 ng/ml epidermal growth factor (EGF) (PeproTech), 0.4% BSA (Sigma-Aldrich), and 5 μg/ml insulin (Sigma-Aldrich). CRC cells were digested and resuspended in the prepared medium. Cells (1 × 10^3^ cells per well) were seeded in 6-well ultralow attachment plates. The cells were grown in the prepared medium in an incubator at 37 °C and 5% CO^2^ with saturated humidity for 12–14 days. The tumor sphere was defined as > 2000 cells. Sphere efficiency was defined as the percentage of the number of spheres divided by the original number of seeded cells. All experiments were conducted at least three times, and the data are presented as the mean ± SD.

### Irinotecan treatment experiment

For the tumor drug treatment assay, 2 × 10^6^ cells per mouse were subcutaneously injected into the right dorsal flanks of female BALB/c athymic nude mice (4–6 weeks of age, 18–20 g), which were obtained from the Animal Center of Southern Medical University, Guangzhou, China). The tumor-bearing mice were observed until the tumor volume reached approximately 150 mm^3^, and the mice were randomly grouped. Mice in the two groups were intraperitoneally injected with 20 mg/kg irinotecan (Selleck, S2217) or DMSO three times per week. The tumor diameters were measured twice a week. Approximately 50 days later, the tumors were excised and placed in 10% neutral buffered formalin for 24 h. For flow cytometry, the indicated cells were seeded into plates with the medium as mentioned above, and 5 μg/ml irinotecan (Selleck, S2217) or PBS was added when the cells adhered to the plates. After 48 h, the cells were detected by flow cytometry according to the manufacturer’s instructions. For tumor sphere formation analysis, a mixture culture medium was prepared as mentioned above and an additional 5 μg/ml irinotecan (Selleck, S2217) or PBS was added. Then, the cells were cultured in these medium in an incubator at 37 °C and 5% CO^2^ with saturated humidity for 12–14 days.

### Statistical analyses

All data were plotted and counted by SPSS19.0 for Windows, represented the mean ± SD. *P* < 0.05 was considered to be statistically significant. The difference in the miR-340 or hsa_circ_001680 expression level between carcinomatous and normal CRC tissues was evaluated by a paired t test. Clinical pathological characteristics of circ_001680 expression in CRC patients were analyzed by a two-sample t test. The linear relationship between circ_001680 and miR-340 expression levels in colorectal cancer cells was measured by Pearson correlation coefficient.

## Results

### Circ_001680 is positively correlated with the occurrence of colorectal cancer

**Fig. 1:**
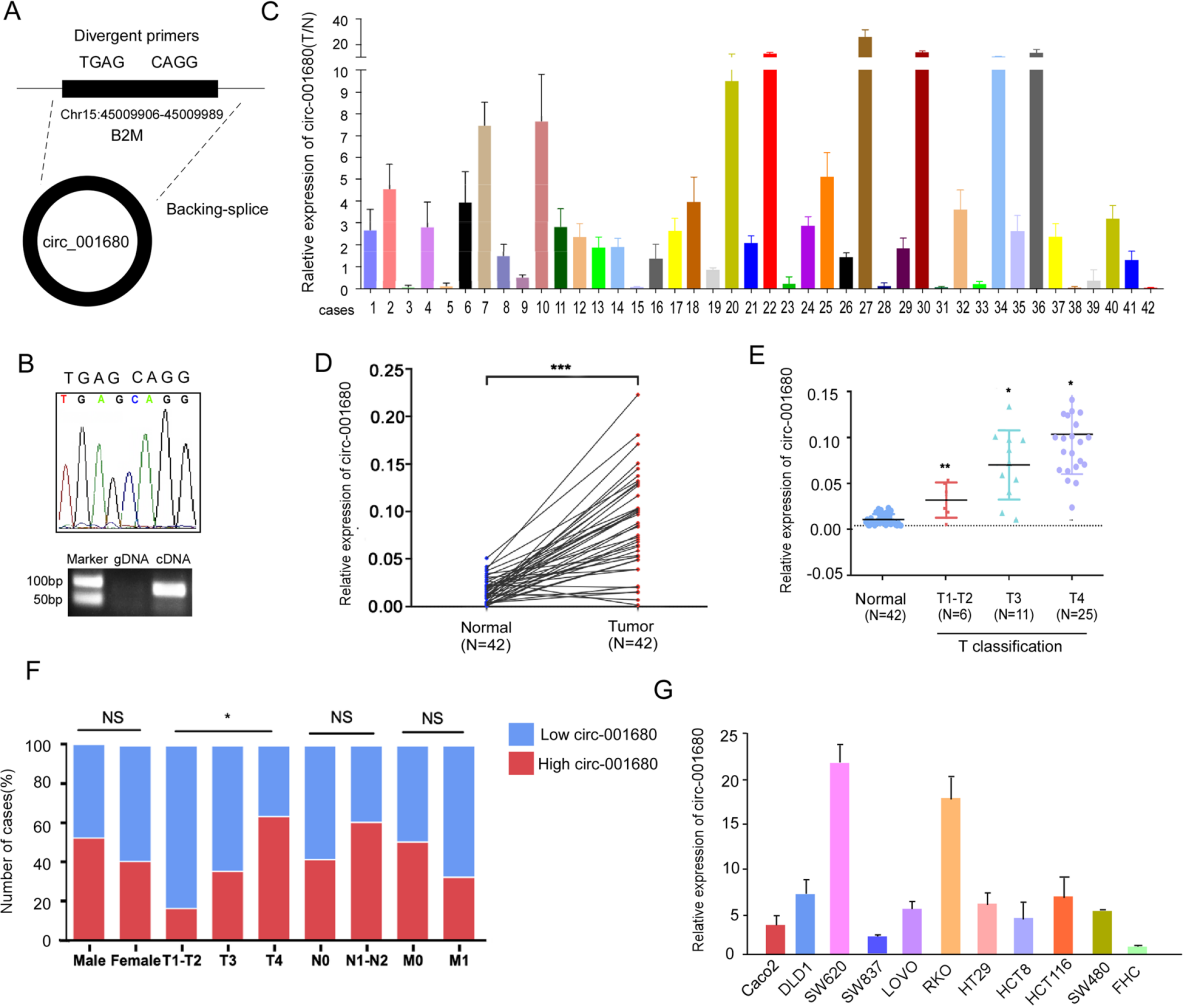
Circ_001680 is overexpressed in colorectal cancer. **a** Circ_001680 is generated from the B2M gene, located on chromosome 15. **b** Upper: Sequencing results showing the back-splice junction sequences of circ_001680. Lower: circ_001680 sequences that were PCR-amplified from cDNA and gDNA with divergent primers, respectively. **c** Relative expression levels of circ_001680 in 42 paired human colorectal cancer tissues. **d** The Mann-Whitney test was used to detect the relative expression level of circ_001680 in 42 normal and noncancerous tissues(***P < 0.005). **e** Correlation between circ_001680 expression and the stage of clinical T classification in 42 cases of CRC and noncancerous tissues (**p < 0.01, *p < 0.05). **f** Graphical illustration of the statistical circ_001680 distribution in CRC patients. (**p < 0.01, *p < 0.05). **g** qRT-PCR analysis of circ_001680 expression in 11 CRC cell lines

Circ_001680 is located at position chr15:45009906–45,009,989, a portion of which is shown in Fig. 1a and Fig. 1b. We designed specific primers spanning the back-splice junction site of circ_001680 (Additional file 1: Table S1).

To assess the influence of circ_001680 on CRC, we examined its expression in 42 pairs of CRC tissues and adjacent normal tissues via qRT-PCR. The results showed that compared to the matched adjacent normal tissues, CRC tissues had circ_001680 overexpression in 71.4% (30/42) of the tissues (T/*N* > 1.5-fold) from the same patients (Fig. 1c). Student’s t test showed that the expression of circ_001680 was significantly higher in the CRC tissue samples than in the adjacent normal tissues (*P* < 0.05, Fig. 1d). Furthermore, the expression of circ_001680 was correlated with the patients’ clinical T stage, as circ_001680 was expressed at relatively low levels in early T stage (T1, T2 and T3) tumors and at significantly increased levels in T4 tumors (*P* < 0.05, Fig. 1e). Then, we divided all CRC patients into high or low circ_001680 expression level groups according to the median value. The results showed statistical circ_001680 distribution in CRC patients based on patient sex and histological classification. As shown in Fig. 1f, higher expression of circ_001680 was significantly correlated with the tumor T stage of colorectal cancer (*P* < 0.05, Fig. 1f). In addition, qRT-PCR analysis was performed to assess the expression of circ_001680 in 11 CRC cell lines, including Caco2, DLD1, SW620, SW837, Lovo, RKO, HT29, HCT8, HCT116, SW480 and FHC (Fig. 1g).

### Circ_001680 promotes the proliferation and migration capacity of colorectal cancer cells

**Fig. 2:**
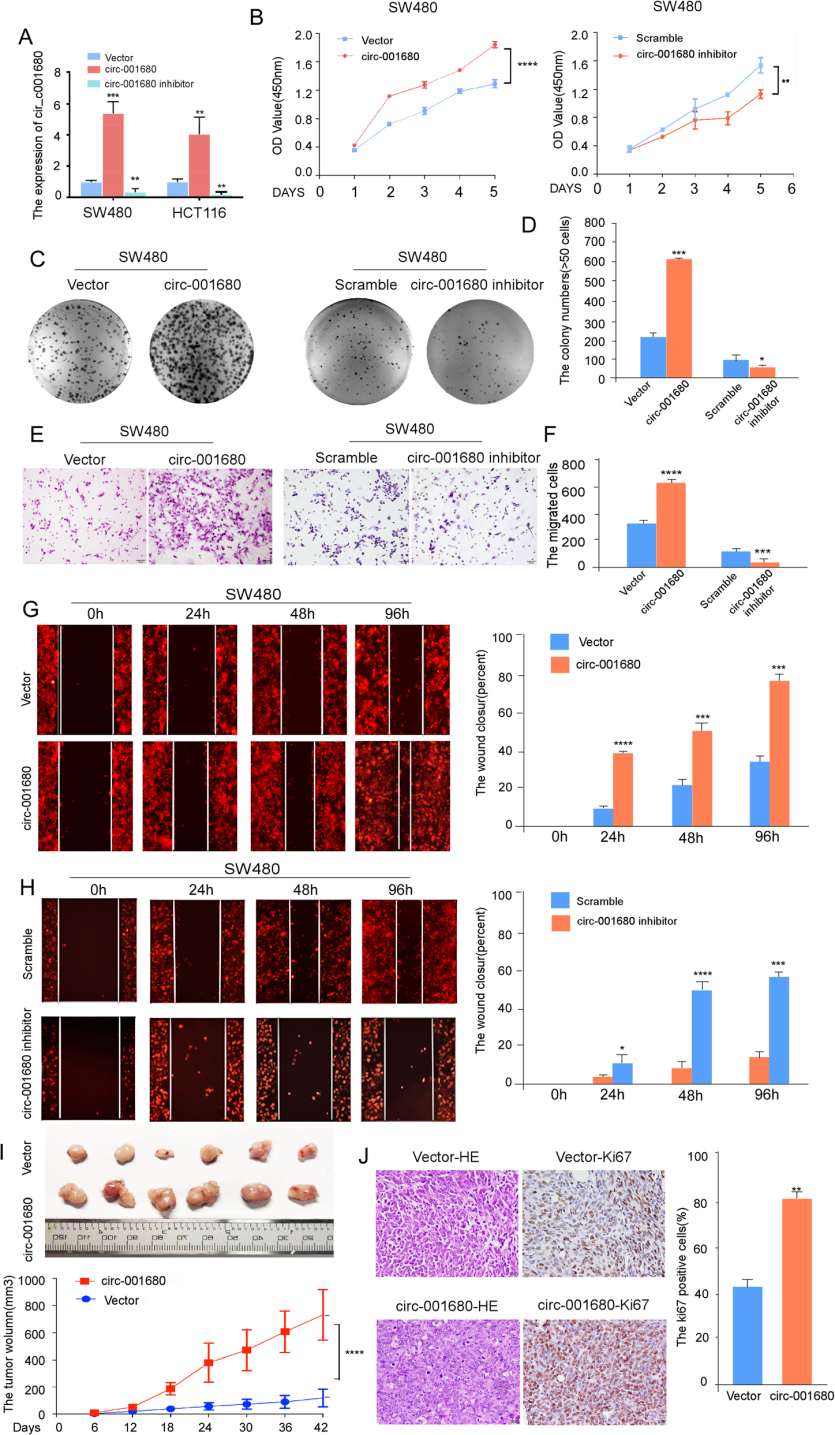
The proliferation and migration of CRC is promoted by circ_001680. **a** Relative expression of circ_001680 in lentivirus-infected SW480 and HCT116 CRC cell lines. **b** CCK8 assay comparing the effects of circ_001680 on cell growth between the SW480-vector and SW480-circ_001680 groups (left) and between the SW480-scramble and SW480-circ_001680 inhibitor groups (right). **c**, **d** Representative images of the colony formation assay in the indicated cells; **d**, statistical analysis of the colony formation results. **e**, **f** E, Representative transwell results comparing the effects of circ_001680 on cell migration between the SW480-vector and SW480-circ_001680 groups (left) and between the SW480-scramble and SW480-circ_001680 inhibitor groups (right); **f**, statistical analysis of the transwell assay results. **g**, **h** Wound healing assay results showing the differences in migration capacities in the indicated cells at 4 regular intervals (left); statistical analysis of the wound healing assay results (right). **i** Image of the tumor xenograft model (upper); differences of tumor volume between the SW480-vector and SW480-circ_001680 cells, which were injected into subcutaneous nude mice (lower). **j** Results of hematoxylin and eosin (H&E) and Ki-67 immunohistochemistry analysis of tumors generated using the tumor xenograft model (left); statistical analysis of Ki-67 positive cells (right). The error bars represent the means±SDs from three independent experiments. **p < 0.01, ***p < 0.005, ****p < 0.001

To investigate whether circ_001680 can affect the function of CRC cells in vitro, we overexpressed and suppressed its expression in SW480 and HCT116 cells using lentiviral delivery, and qRT-PCR analysis showed that stable cell lines were successfully constructed (Fig. 2a). As shown in Fig. 2b, c, d and Additional file 2: Figure S1, the results of CCK8 assays and colony formation revealed that the overexpression of circ_001680 in SW480 and HCT116 cells promoted their proliferative capacity. Subsequently, transwell migration and wound healing assays were performed to assess the influence of circ_001680 on the migration of CRC cells. As expected, circ_001680 overexpression remarkably enhanced the migration capacity of both SW480 and HCT116 cells (Fig. 2e, f, g and Additional file 3: Figure S2). However, this phenomenon was remarkably reversed when circ_001680 was inhibited (Fig. 2b-h).

Consistent with the in vitro findings, overexpression of circ_001680 increased subcutaneous xenograft growth in vivo (Fig. 2i). CRC cells that stably overexpressed circ_001680 were injected into the dorsal subcutaneous tissues of 6 nude mice, and as shown in Fig. 2i, the volumes of the tumors formed by circ_001680-overexpressing CRC cells grew faster than those formed by control cells. Furthermore, the percentage of ki67-positive cells in subcutaneous tumors was higher in the circ_001680 group than in the control group (Fig. 2j). The volume of the tumor was significantly smaller in the circ_001680 inhibited group than in the control group (Additional file 2: Figure S1D and E). Taken together, these results reveal that circ_001680 enhances the proliferation and tumorigenicity of CRC cells.

### Circ_001680 directly targets the miR-340 to suppress its transcriptional regulatory activity

**Fig. 3:**
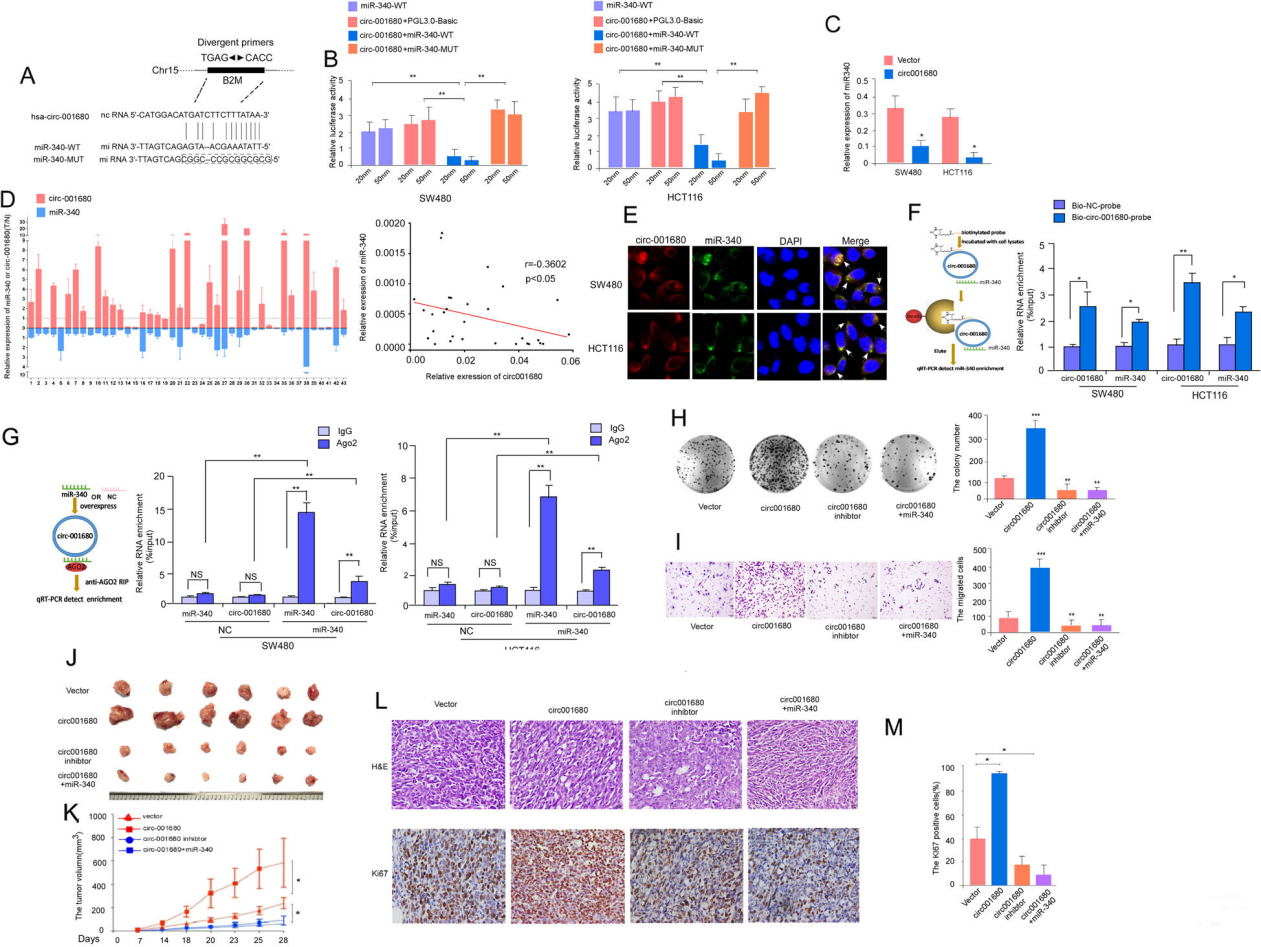
Circ_001680 targets miR-340 and inhibits its transcriptional regulatory activity. **a** Graphical illustration showing the predicted position of the circ_001680 target on the miR-340 sequence. **b** Luciferase activity assay showing that circ_001680 influences the luciferase activity of miR-340 3’UTRs in SW480 and HCT116 cells. **c** qRT-PCR analysis of miR-340 in the indicated lentivirus-infected cells. **d** qRT-PCR analysis of circ_001680 and miR-340 expression in 43 human colorectal cancer tissues (left); correlation analysis showing that the expression of circ_001680 is negatively correlated with miR-340 (right). **e** The co-localization between circ_001680 (red) and miR-340 (green) was observed (arrowheads) by fluorescence in situ hybridization in SW480 and HCT116 cells. Cell nuclei were counterstained with DAPI (blue). Scale bar, 5 μm. **f** According to the RNA pull-down flowchart (left), whole-cell lysates from SW480 and HCT116 cells were incubated with biotinylated probes against circ_001680 or NC; after pull-down, endogenous circ_001680 and miR-340 enrichments were detected by qRT-PCR. Results were presented as the percentage of pull-down to input. **g** According to the flowchart outlining the experimental procedures (left), The complex containing circ_001680 and miR-340 in indicated cells was immunoprecipitated by anti-Ago2 using RIP (RNA immunoprecipitation) assay and followed by qRT-PCR analyses to detect circ_001680 and miR-340 enrichment (right). **h**, **i** Overexpression of miR-340 inhibits the cell growth and migration capacities of SW480 cells induced by circ_001680, as determined by a colony formation assay (**h**) and a transwell assay (**i**) in SW480 cells. **j** Image of the subcutaneous tumor mode. SW480-vector, SW480-circ_001680, SW480-circ_001680 inhibitor and SW480-circ_001680 + miR-340 cells were subcutaneously injected into nude mice separately (*n* = 6/group). **k** The tumor volume among the four indicated groups at the indicated time points. **l** H&E (upper) and Ki-67 immunohistochemistry (IHC, lower) assays were adapted to detect the tumor section. **m** Statistical analysis of Ki-67-positive cells. All tests were carried out in triplicate. The error bars represent the means ± SDs from three independent experiments. *p < 0.05**, *p* < 0.01, ***p < 0.005

We used the bioinformatics website starBase (http://starbase.sysu.edu.cn/index.php) to predict that circ_001680 could bind to miR-340, and the predicted binding sites within circ_001680 and miR-340 were shown in Fig. 3a. The pre-experiment results showed that circ_001680 had a negative correlation with miR-340 in the 20 fresh colon tissues (*r* = − 0.7350, *p* < 0.005, Additional file 6: FigureS5). Subsequently, we observed a surprising reduction in luciferase activity in cells co-transfected with the circ_001680 and miR-340-wild-type (WT) reporter genes (Fig. 3b). Furthermore, qRT-PCR analysis showed that miR-340 expression was significantly downregulated in cells overexpressing circ_001680, whereas its expression was notably increased in circ_001680-downregulated cells (Fig. 3c). To confirm the relationship between miR-340 and circ_001680 in CRC, we detected their expression in the same 43 paired CRC tissues by real-time RT-PCR, revealing that circ_001680 was negatively correlated with miR-340 (*r* = − 0.3602, *p* < 0.05, Fig. 3d). In addition, we performed FISH experiments to detect the relationship between circ_001680 and miR-340. The FISH results showed that circ_001680 and miR-340 were preferentially colocalized in the cytoplasm (Fig. 3e). RNA pull-down analysis demonstrated that endogenous miR-340 was significantly pulled down by biotinylated probes against circ_001680 (Fig. 3f). Furthermore, the anti-AGO2 RIP results showed that endogenous circ_001680 pull-down by AGO2 was specifically enriched in SW480 and HCT116 cells upon overexpression of miR-340 (Fig. 3g), validating the direct binding of circ_001680 with miR-340.

Next, colony formation and transwell migration assays were performed to assess whether miR-340 could reverse the effect of circ_001680 on the proliferation and migration of CRC cells. As shown in Fig. 3h and i, the overexpression of miR-340 inhibited the “oncogenic” effect of circ_001680 in CRC cells.

To further assess the in vivo effects of miR-340 on diminishing the “oncogenic” effect of circ_001680 in CRC cells, SW480/vector, SW480/circ_001680, SW480/circ_001680 inhibitor or SW480/circ_001680 + miR-340 cells were subcutaneously injected into nude mice (*n* = 6 for each group). As shown in Fig. 3j and k, large subcutaneous tumors were observed in mice of the SW480/circ_001680 group, whereas the tumors in mice of the SW480/circ_001680 inhibitor and SW480/circ_001680 + miR-340 cell groups were remarkably smaller (Fig. 3k). Notably, the percentages of Ki67-positive cells were notably reduced in the SW480/circ_001680 inhibitor and SW480/circ_001680 + miR-340 groups (Fig. 3l and m). Taken together, these results indicate that circ_001680 can affect the function of CRC cells by targeting miR-340 both in vivo and in vitro.

### Circ_001680 affects the expression of BMI1 by targeting miR-340

**Fig. 4:**
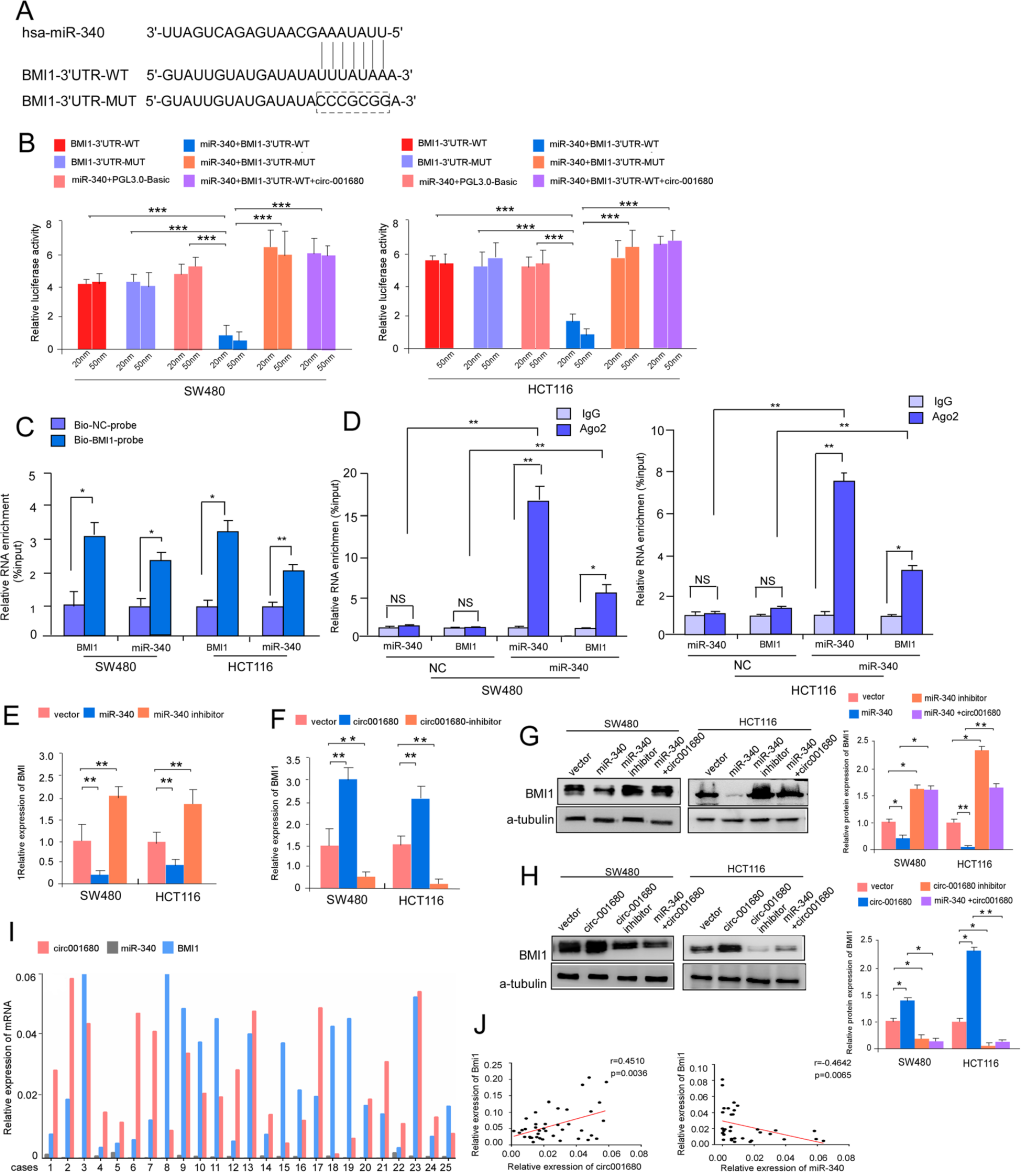
Circ_001680 binds to miR-340 to suppress the expression of BMI1. **a** Construction of BMI1 3′-UTR WT and mutant (Mut) luciferase reporter vectors. **b** The dual-luciferase reporter assays indicated that luciferase activity was decreased in CRC cells after cotransfection with the BMI1–3′-UTR-WT and miR-340 mimics. **c** The whole-cell lysates from SW480 and HCT116 cells were incubated with biotinylated probes against BMI1 or NC; after pull-down, endogenous BMI1 and miR-340 enrichments were detected by qRT-PCR. Results were presented as the percentage of pull-down to input. **d** The complex containing miR-340 and BMI1 in SW480 and HCT116 cells was immunoprecipitated by by anti-Ago2 using RIP (RNA immunoprecipitation) and followed by qRT-PCR analyses to detect miR-340 and BMI1 enrichment. All tests were carried out in triplicate. **e**, **f** qRT-PCR analysis of BMI1 in the indicated cells. **g**, **h** Protein expression of BMI1 in the indicated cells as determined by Western blotting. α-tubulin was used as a loading control. The protein expression levels were quantified by comparing the gray level of each band using Quantity One Software. **i** qRT-PCR analysis of circ_001680, miR-340 and BMI1 expression in the 25 fresh human CRC samples. **j** Spearman correlation analyses between BMI1 and circ_001680 expression (left), as well as BMI1 and miR-340 (right) expression in 25 fresh human CRC samples (*p* < 0.01).

Evidence has shown that circRNAs sequester miRNAs to terminate the regulation of their target genes [27–29]; thus, we speculated that circ_001680 could affect the target gene of miR-340. The bioinformatics algorithm miRbase (http://www.mirbase.org/) predicted that BMI1 was the target gene of miR-340. Western blot assays revealed the expression of BMI1 was correlated negatively with miR-340(Additional file 4: Figure S3A). Consistent with the predicted results, ectopic upregulation of BMI1 reversed the influence of miR-340 on CRC cell growth (Additional file 4 Figure S3B and C) and migration (Additional file 4: Figure S3D). We cloned the 3′UTR fragments of BMI1 containing the miR-340 binding sites and 3′UTR mutant fragments into the pGL3-basic luciferase reporter vectors separately (Fig. 4a). Moreover, a consistent reduction in luciferase activity was observed upon miR-340 and BMI1–3’UTR-WT co-transfection in both SW480 and HCT116 lines (Fig. 4b). Interestingly, the luciferase activity was recovered after transfection with circ_001680 in the miR-340 + BMI1–3’UTR-WT cell line. However, BMI1–3’UTR mutations abrogated the suppressive effect. Furthermore, an RNA pull-down assay was performed to detect the relationship between miR-340 and BMI1. The analysis demonstrated that endogenous BMI1 was significantly pulled down by biotinylated probes against miR-340 (Fig. 4c). We used anti-AGO2 RIP in SW480 and HCT116 cells and result showed miR-340 pull-down by BMI1 was specifically enriched in SW480 and HCT116 cells (Fig. 4d). In addition, ectopic miR-340 expression in SW480 and HCT116 cells reduced the mRNA and protein levels of BMI1, while knockdown of miR-340 led to increased expression of BMI1 (Fig. 4e and g). However, cotransfection with circ_001680 reversed the expression of BMI1 in the miR-340 overexpression group (Fig. 4g). Western blot assays were performed to explore whether circ_001680 affects the expression of BMI1, revealing that circ_001680 increased the protein and mRNA levels of BMI1 in CRC cells, while knockdown of circ_001680 reversed these effects (Fig. 4f and h). As predicted, the expression of BMI1 was decreased after the cotransfection of miR-340 in the circ_001680 group (Fig. 4f and h). In addition, we analyzed the expression of circ_001680, miR-340 and BMI1 in 25 CRC tissue samples to assess their relationships (Fig. 4i). Figure 4j shows that circ_001680 and BMI1 were positively correlated (*r* = 0.4510, *p* = 0.0036), whereas BMI1 was negatively correlated with the expression of miR-340 (*r* = − 0.4642, *p* < 0.0065). These results confirm that as a miR-340 sponge, circ_001680 not only affects the expression of miR-340 but also upregulates its target gene BMI1.

### Circ_001680 promotes the CSC population in CRC

**Fig. 5:**
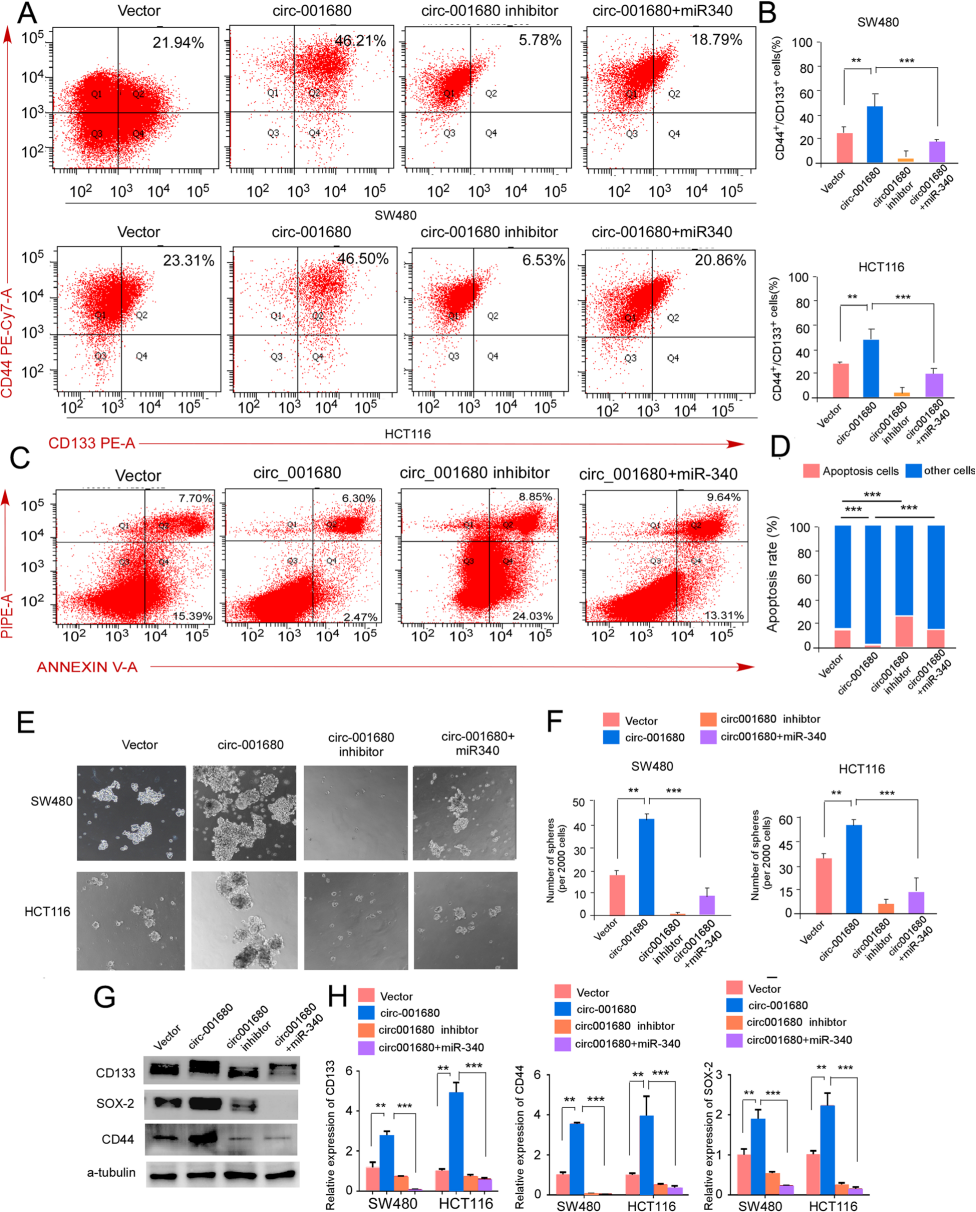
Circ_001680 enhances the colorectal cancer CSC population in vitro. **a** The number of subpopulations with the CD44+/CD133+ phenotype in the indicated SW480 (upper) and HCT116 (lower) cells. **b** Statistical results of the number of subpopulation cells with the CD44+/CD133+ phenotype. **c** The apoptosis assay conducted by flow cytometry in the indicated group. Flow cytometry analyses of SW480 cells treated with 1.0 μM doxorubicin for 24 h. **d** Statistical analysis of flow results. Annexin-positive/PI-negative cells were analyzed to determine the apoptosis rate. Error bars represent the mean ± SD from three independent experiments. **e** Typical images from the sphere formation assay among vector, circ_001680, circ_001680 inhibitor and circ_001680 + miR-340. **f** Graphical illustration showing the sphere formation efficiency from Figure 5C. **g** Western blot analysis of CD133, SOX2 and CD44 in the indicated lentivirus-infected cells. **h** qRT-PCR analysis of CD133, CD44 and SOX2 in the indicated cells.

BMI1 has been reported to be a positive regulator that induces the stem cell-like properties of cancer cells [30–32]. Given these results, we suspected that circ_001680 may also regulate the stem cell-like characteristics of CRC cells, and we thus investigated the role of circ_001680 in the maintenance of the CSC-like phenotype. Accumulating research indicates that cancer cells expressing the surface marker phenotype CD44+/CD133+ have stem cell-like traits and possess self-renewal and tumor-initiating capacity [30]. The results of a flow cytometry-based analysis showed that the double positive population (CD44+/CD133+) was increased in the circ_001680 overexpression group, whereas the proportion of CD44+/CD133+ was decreased in the circ_001680 inhibition group (Fig. 5a-b). The number of apoptotic cells was reduced in circ_001680 group and the proportion of apoptotic cells was increased in the circ_001680 inhibition and circ_001680 + miR-340 group (Fig. 5c-d). Interestingly, when circ_001680 and miR-340 were both upregulated, the high proportion of CSC markers was reversed. Similarly, the results of the sphere formation assay demonstrated that the circ_001680-overexpressing cells could form more viable spheres, whereas the number of spheres was significantly reduced through the upregulation of miR-340 in circ_001680-overexpressing cells (Fig. 5e and f). Additionally, Fig. 5g and h demonstrated that the mRNA and protein levels of SOX2, CD44, and CD133 were increased when circ_001680 was upregulated. However, the expression of these stem cell markers was decreased in the circ_001680 inhibitor and miR-340 + circ_001680 group.

### Circ_001680 induces irinotecan therapeutic resistance in CRC cells

**Fig. 6:**
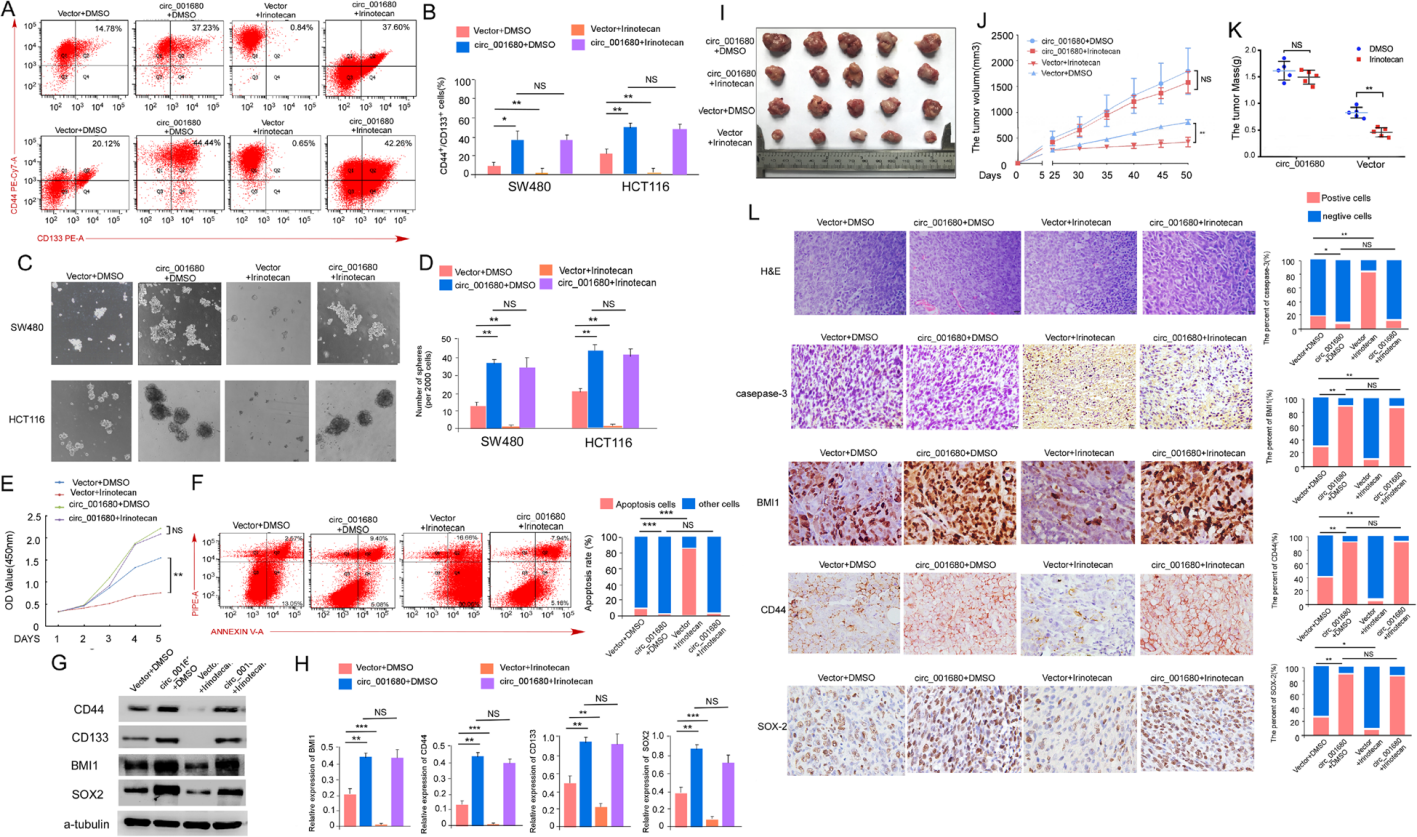
Overexpression of circ_001680 suppresses the sensitivity of CRC cells to irinotecan. **a** The numbers of subpopulation cells with the CD44+/CD133+ phenotype in the indicated SW480 (upper) and HCT116 (lower) cells. **b** Quantification of cells with the CD44+/CD133+ phenotype is shown in the histogram (*P < 0.05, **P < 0.01). **c** Typical images from the sphere formation assay in the indicated lentivirus-infected cells treated with or without irinotecan. **d** Bar graph showing the sphere formation efficiency (*P < 0.05, **P < 0.01). **e** CCK8 analyzed cell growth capacity in the indicated cells (**P < 0.01). **f** Apoptosis assay of the indicated cells by flow cytometry (middle). Statistical analysis of the flow cytometry results (right). **g** Western blot analysis of CD44, CD133, BMI1 and SOX2 in the indicated cells. **h** qRT-PCR analysis of BMI1, CD44, CD133 and SOX2 in the indicated cells. **i** Images of the tumor xenograft model (*n* = 5). **j** Tumor volume statistics among the four indicated groups on the indicated days. **k** The tumor weight statistics among the four indicated groups. **l** H&E and immunohistochemistry analyses of xenograft tumors. IHC staining was performed using antibodies against caspase-3, BMI1, CD44 and SOX-2. The error bars represent the means±SDs from three independent experiments (*P < 0.05, **P < 0.01)

Irinotecan is a semisynthetic derivative of camptothecin that exerts cytotoxicity through topoisomerase and is used as the primary chemotherapeutic drug to treat metastatic CRC. However, the drug resistance of advanced CRC gradually reduces the effectiveness of this drug [33]. CSCs have been considered to be the primary cause of chemotherapy resistance [34–36]. In previous experiments, we confirmed that circ_001680 promoted the CSC population in CRC. Next, we assessed whether circ_001680 induces irinotecan therapeutic resistance, and flow cytometry analysis showed that the proportion of CD44+/CD133+ cells had no significant change in the circ_001680-overexpressing group after irinotecan treatment. However, the number of CD44+/CD133+ cells was remarkably decreased after treatment with the drug in the control group. (Fig. 6a and b). In a sphere formation assay, SW480-circ_001680 and HCT116-circ_001680 were shown to form more stem cell spheres than the control group after treatment with irinotecan (Fig. 6c and d). The CCK8 analysis showed the cell growth ability was stronger in circ_001680 group after treatment with the drug (Fig. 6e). As shown in Fig. 6f, the number of apoptotic cells was increased after treatment with irinotecan in the control group. However, this effect was abrogated when circ_001680 was upregulated. Similarly, the mRNA and protein levels of BMI1, CD133, CD44, and SOX-2 were remarkably higher in the circ_001680-overexpressing group than in the control group after treatment with irinotecan (Fig. 6g and h). Interestingly, there was no significant change in the subcutaneous tumor volumes and weights after the intraperitoneal injection of irinotecan in the circ_001680-overexpressing group (Fig. 6i-k). However, the tumor sizes and weights were remarkably decreased after treatment with drugs in the control group (Fig. 6i-k).

Furthermore, the expression of stem cell markers on tumor sections was not significantly decreased in the circ_001680-overexpressing group (Fig. 6l). These results indicated that circ_001680 induced irinotecan therapeutic resistance in CRC cells.

## Discussion

In recent years, an increasing number of studies have identified circRNAs that may serve as diagnostic or predictive biomarkers of some diseases, especially cancers [37, 38]. For instance, hsa_circ_0013958 may be useful as a potential noninvasive biomarker for the early detection and screening of lung adenocarcinoma (LAC) [39]. Circ_0026344 acts as a prognostic biomarker to suppress colorectal cancer progression via microRNA-21 and microRNA-31 [40]. CircPVT1 is significantly overexpressed in osteosarcoma (OS) tissues, serum, and chemoresistant cell lines, suggesting that this circRNA is a potential diagnostic biomarker with useful sensitivity and specificity [41].

In our research, functional experiments showed that circ_001680 overexpressing cell lines could increase the cellular capabilities of proliferation and migration. At the tissue level, we used qRT-PCR to assess the expression of circ_001680 in human CRC tissues and their matched normal tissues, revealing that circ_001680 was expressed at higher levels in CRC tissues than in their matched normal tissues. Bioinformatics predictions and the dual-luciferase reporter experiments demonstrated that circ_001680 could target miR-340, which has been identified as a miRNA that is downregulated in many types of cancer. MiR-340 can inhibit cell proliferation, induce cell apoptosis, and reduce cell migration and invasion by targeting multiple oncogenes in breast cancer, gastric cancer, glioblastoma, and non-small-cell lung cancer. Bioinformatics predictions and the luciferase reporter system results showed that miR-340 could target the 3’UTR of BMI1 and that ectopic upregulation of BMI1 partially reversed the influence of miR-340 on CRC cell growth and migration (Additional file 4: Figure S3).

BMI1 is a core component of the polycomb repressive complex that mediates gene silencing via monoubiquitination of histone H2A [42, 43]. Furthermore, BMI1 is an important stem cell self-renewal factor [44, 45] that has been shown to be abnormally expressed in CRC and is associated with the self-renewal of CSCs in CRC [46]. Targeting BMI1^+^ CSCs has been shown to overcome chemoresistance and inhibit metastases in squamous cell carcinoma [47] and gastric cancer [32]. Liu et al. found that overexpression of miR-128-3p could reestablish sensitivity in resistant cells by reducing BMI1 expression, which is related to oxaliplatin resistance [48]. CSCs are believed to function as a type of stem cell-like cell population in tumors that promote self-renewal [49], and they are associated with tumor metastasis [50, 51] and drug resistance [34, 52]. In our research, we found that BMI1 could induce irinotecan chemotherapy resistance in CRC cells. We transiently transfected cells with BMI1 at different concentrations in the cells, and the results showed that higher concentrations of BMI1 were correlated with more obvious chemotherapy resistance effects (Additional file 5: Figure S4). Although irinotecan is known to be effective for patients with advanced CRC, chemotherapy resistance to the drug often leads to cancer treatment failure. It is necessary to investigate the biological basis of chemotherapy resistance and identify an effective marker.

Thus, we suspected that circ_001680 could affect the stem cells characteristics of CRC and induce chemoresistance by upregulating the expression of BMI1. There were no significant changes in the number of stem cell spheres in the circ_001680 group after treatment with irinotecan, whereas a notable decrease was observed in the control group in response to irinotecan treatment. Similarly, the levels of stem cell markers and the populations of CD133+/CD44+ cells were remarkably decreased in the control group compared with the levels in the circ_001680 group after treatment with irinotecan. We subcutaneously injected the indicated CRC cells into female BALB/c athymic nude mice. When the tumor volume reached approximately 150 mm^3^, mice were intraperitoneally injected with 20 mg/kg irinotecan or DMSO three times per week. Interestingly, the tumor volumes and weights of the mice in the drug groups were notably smaller than those in the DMSO group. However, upregulation of circ_001680 did not result in significantly different volumes and weights in the drug and DMSO groups. These findings demonstrate a novel role for circ_001680 in the regulation of stem cell characteristics and chemoresistance and provide a molecular basis for targeting BMI1 to overcome irinotecan chemoresistance in colon cancer.

Our study demonstrated that circ_001680 could mediate CRC tumor growth and migration for the first time. At the same time, we found an essential link connecting circ_001680, miR-340 and BMI1 in CRC. Furthermore, circ_001680 was shown to promote the CSC population in CRC and induce irinotecan chemoresistance by upregulating the miR-340 target gene BMI1. Understanding the precise function and mechanism of circ_001680 in the progression of CRC will increase our understanding of CRC biology and may also allow the development of a novel irinotecan chemoresistance therapeutic strategy.

## Conclusion

In summary, we established a previously unknown function for circ_001680 in CRC. The effects of circ_001680 on cell proliferation and migration suggest that circ_001680 promotes the tumorigenesis and progression of CRC. We also provide evidence that circ_001680 promotes the CSC population in CRC and induces irinotecan therapeutic resistance by regulating the miR-340 target gene BMI1. This finding suggests that circ_001680 is a potential diagnostic biomarker and has the potential to be developed into a new screening method and new chemotherapy resistance target for CRC.

### Supplementary information

**Additional file 1: Table S1.** The primer sequences used for real-time quantitative PCR.

**Additional file 2: Figure S1.** Circ_001680 affected the proliferation ability of CRC cells in vitro. (A) CCK8 assay results comparing the effects of circ_001680 on cell growth between the HCT116 vector and HCT116 circ_001680 groups (left) and between the HCT116 scramble and HCT116 circ_001680 inhibitor groups (right). (B) Representative images of colony formation in the indicated cells. The colonies containing > 50 cells were scored. (C) The number of colonies in an entire well was counted. The error bars represent the mean ± SD from three independent experiments (**p < 0.01, ***p < 0.005). (D) Image of the tumor xenograft model (*n* = 6). (E) Tumor growth curve. The error bars represent the means ± SD from three independent experiments (***p < 0.005).

**Additional file 3: Figure S2.** Circ_001680 affected the growth ability of CRC in vitro. (A) Representative transwell images of the effect of circ_001680 on the migration of the indicated cells (left). Statistical analysis of the transwell assay results (right). (B) The wound healing assay results showing divergent migration capacities at 4 regular intervals in the indicated cells (left); the statistical analysis is shown on the right. The error bars represent the means ± SDs from three independent experiments. *p < 0.05, **p < 0.01, ***p < 0.005.

**Additional file 4: Figure S3.** BMI1 is the bona fide effector of miR-340 in vivo. (A) Western blot analysis of BMI1 in the indicated cells. (B) Representative growth of the indicated cells as determined by the CCK8 assay. (C) Representative images (left) and statistical chart (right) of the colony formation assay in the indicated cells. (D) Representative images (left) and statistical chart (right) of migrated cells across the transwell chamber in indicated cells. The error bars represent the means ± SD from three independent experiments. *p < 0.05, **p < 0.01.

**Additional file 5: Figure S4.** Irinotecan resistance induced by different concentration gradients of BMI1 in CRC cells. (A) SW480 cells were transiently transfected with the indicated amounts of BMI1. The protein level of BMI1 was detected by Western blotting after 48 h. (B) Representative growth of the indicated cells as determined by a CCK8 assay. (C) The number of subpopulation cells with the CD44+/CD133+ phenotype in the indicated SW480 cells (left). Quantification of cells with the CD44+/CD133+ phenotype is shown in the histogram (right). (D) Apoptosis assay of the indicated cells by flow cytometry (left). Statistical analysis of the flow cytometry results (right). (E) Typical images from the sphere formation assay of the indicated lentivirus-infected cells treated with or without irinotecan. The error bars represent the mean ± SD from three independent experiments. **p < 0.01, ***p < 0.005, ****p < 0.001.

**Additional file 6: Figure S5.** circ_001680 was negatively correlated with miR-340. (A)qRT-PCR analysis of circ_001680 and miR-340 expression in 20 fresh human colorectal cancer tissues. (B) Correlation analysis showed that the expression of miR-340 is negatively correlated with circ_001680.

## Data Availability

All data generated or analysed during this study are included in this published article and its Additional files.

## Abbreviations

BMI1: B cell-specific Moloney murine leukemia virus integration site 1
CCK8: Cell Counting Kit-8
circRNA: circular RNA
CRC: Colorectal cancer
CSC: Cancer stem cell
miRNA: microRNA
