# Correction: Hsa_circ_001680 affects the proliferation and migration of CRC and mediates its chemoresistance by regulating BMI1 through miR-340

**DOI:** 10.1186/s12943-024-02018-7

**Published:** 2024-05-14

**Authors:** Xiangyu Jian, Han He, Jiehong Zhu, Qi Zhang, Zhongxin Zheng, Xiangjing Liang, Liuyan Chen, Meiling Yang, Kaiyue Peng, Zhaowen Zhang, Tengfei Liu, Yaping Ye, Hongli Jiao, Shuyang Wang, Weijie Zhou, Yanqing Ding, Tingting Li

**Affiliations:** 1 Department of Pathology, Nanfang Hospital, Southern Medical University, Guangzhou, 510515 Guangdong China; 2 Department of Pathology, School of Basic Medical Sciences, Southern Medical University, Guangzhou, Guangdong China; 3 Department of Hematology, Nanfang Hospital, Southern Medical University, Guangzhou, Guangdong China

**Correction** **:** **Mol Cancer 19, 20 (2020)**

**https://doi.org/10.1186/s12943-020-1134-8**

Following publication of the original article [1], the authors would like to request for the below changes.

1. We request to replace the misused image in Fig.2H-Scramble-96h with the correct image.

Figure 2H


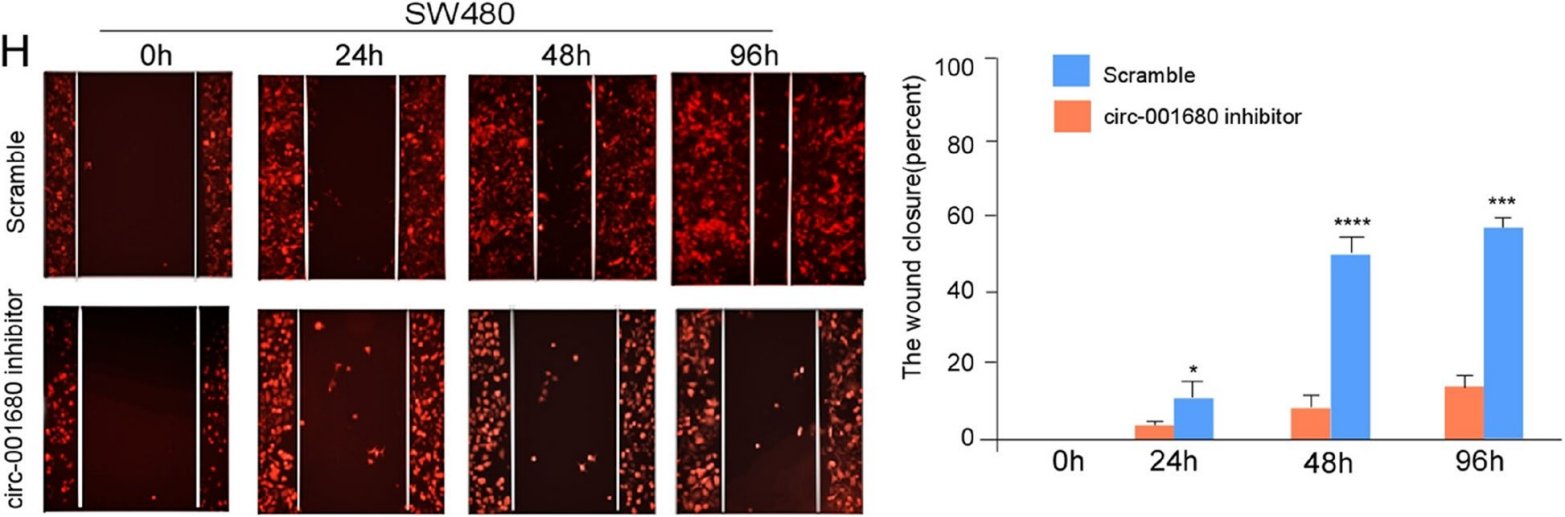


2. We request to replace the misused images of Fig. 3I-circ001680+miR340 and 3L-ki67- circ001680+miR340 with the correct images.

Figure 3I and 3L


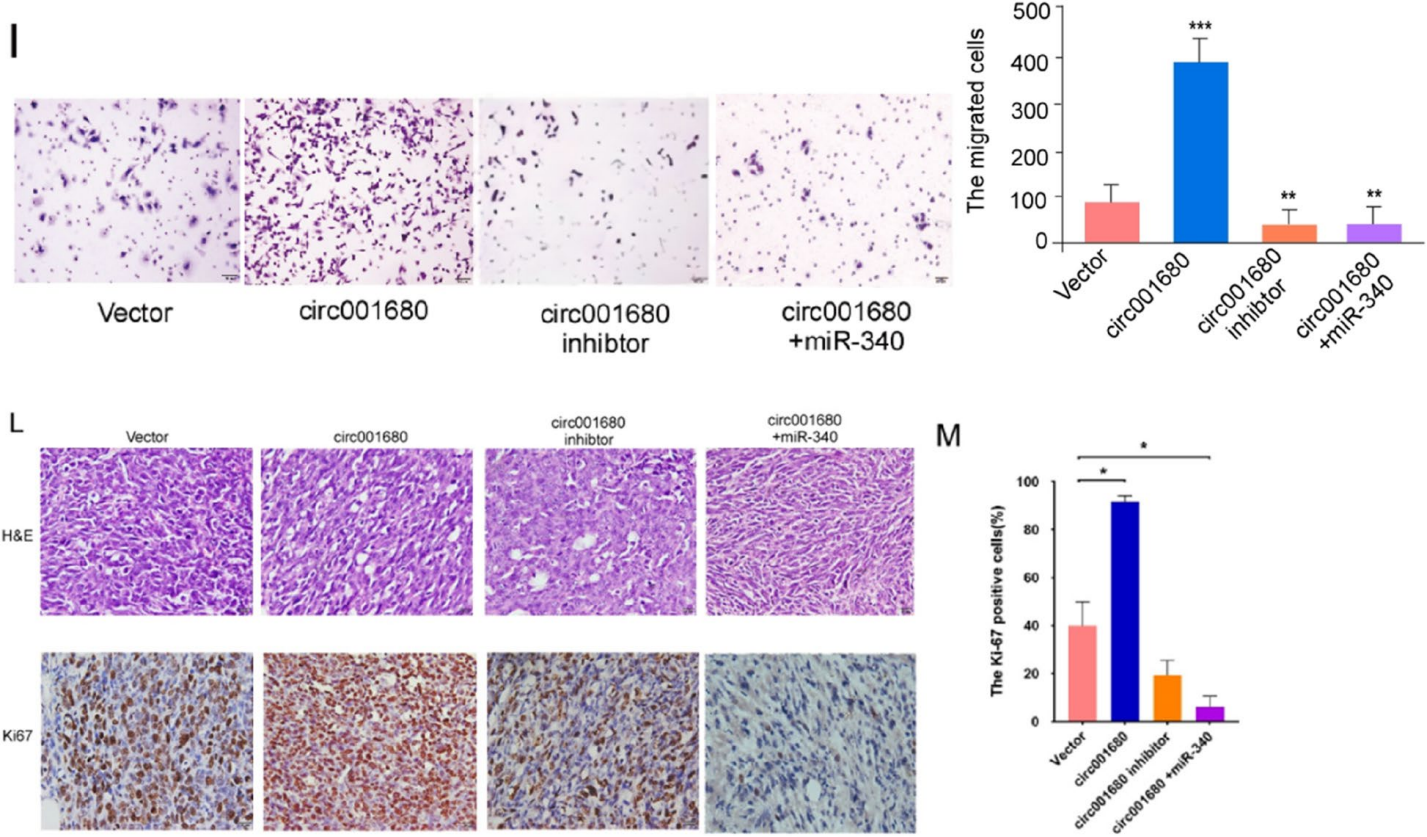


3. We request to replace the misused images in Figure S2A-Vector, Figure S2B and 2C with the correct images.


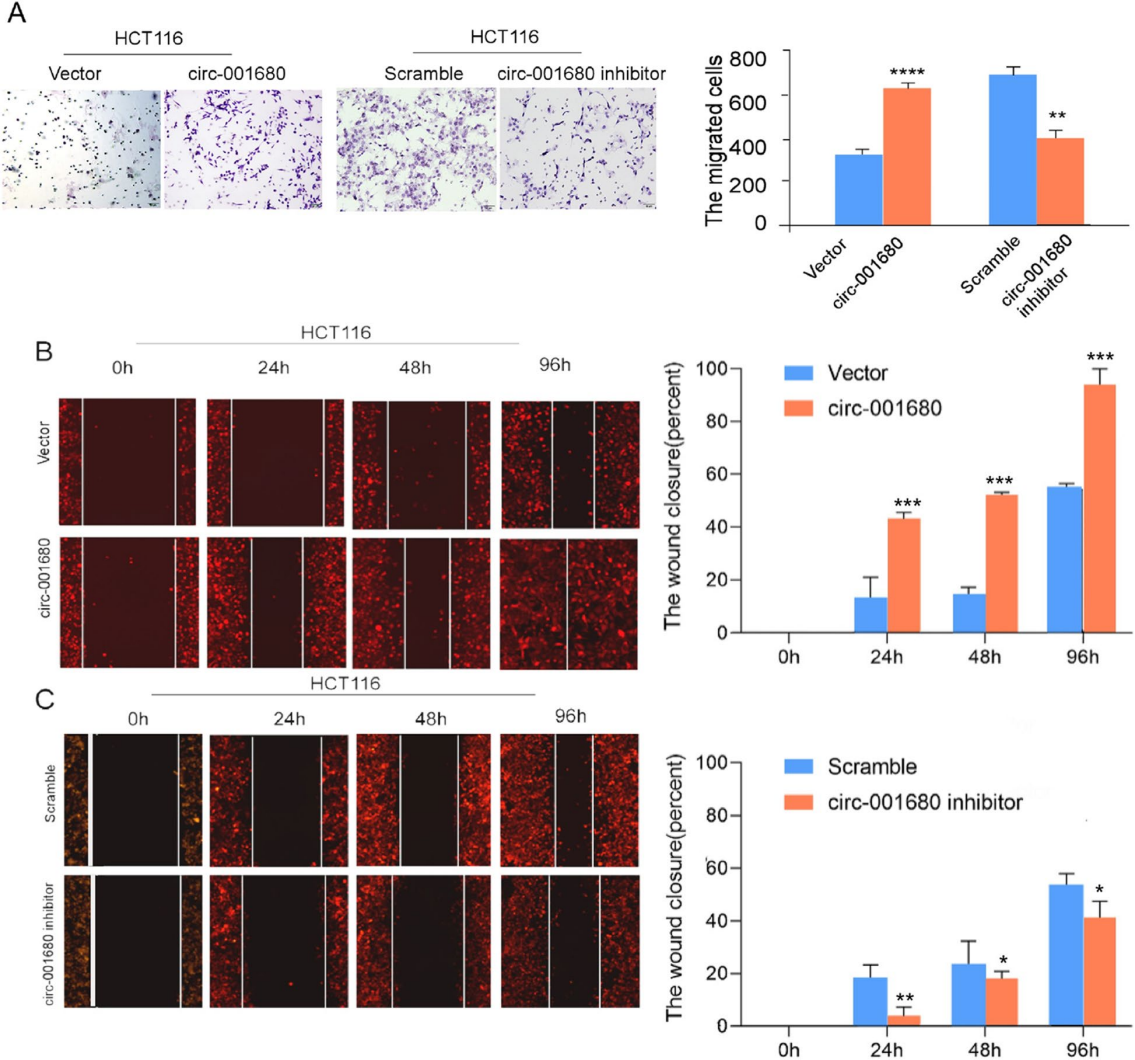


4.We request to replace the misused images in the Figure S4A-a-tublin and S4E-0ug+DMSO with the correct images.

Supplementary Figure S4A and 4E


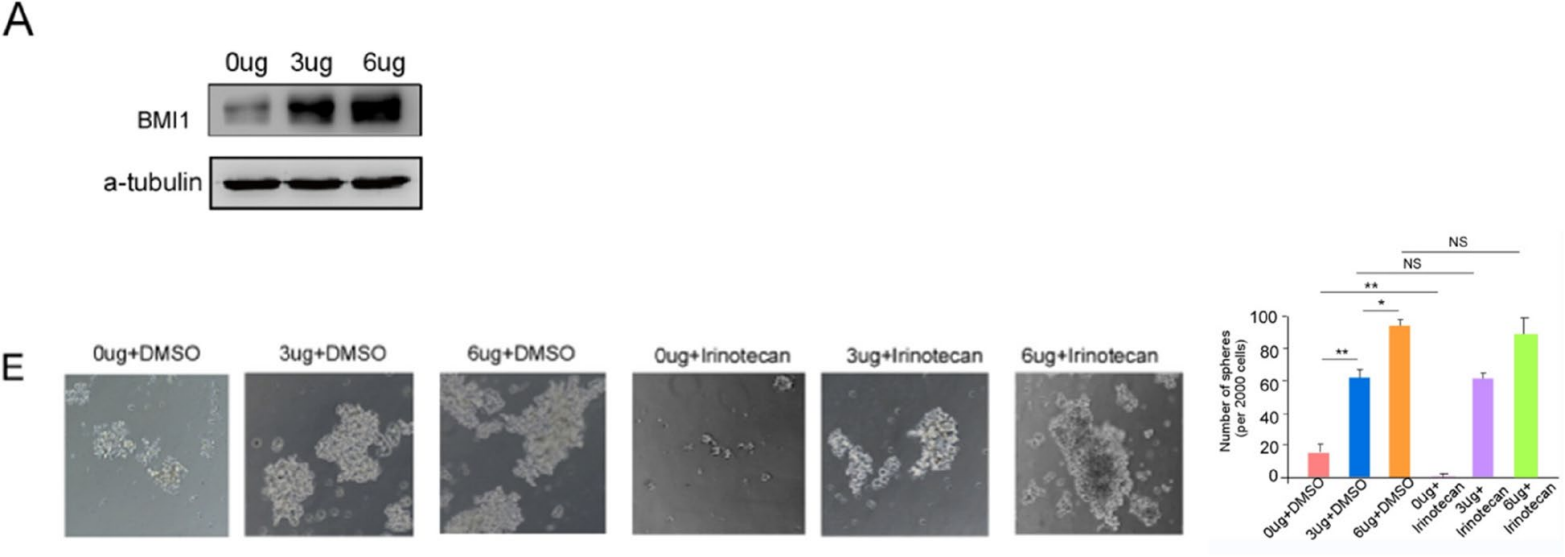


5. We request to replace the sequences in Supplementary Table 1 of miR-340 and circ_001680.

**Table:** 

miR-340	F:ACACTCCAGCTGGGTTATAAAGCAATGAGACT	R:CTCAACTGGTGTCGTGGAGTCGGCAAGAGTCGGCAATTCAGTTGAGAATCAGTCTCAT
Bio-circ_001680-probe	5’Bio-TATAACCCTGCTCAGATACATCAAAC-3′-Bio	
Bio-miR-340-probe	5’BIO-AATCAGTCTCATTGCTTTATAA- 3’BIO	
Digo-circ_001680-probe	5’Digo-TATAACCCTGCTCAGATACATCAAAC-3′-Digo	

The correction does not change the results and scientific conclusions of this article. We sincerely apologize to the editor, reviewers and readers for the errors and any confusion it may have caused.
