# Supplementary material for: Hsa_circ_001680 affects the proliferation and migration of CRC and mediates its chemoresistance by regulating BMI1 through miR-340
Source: Mol Cancer. 2020 Jan 31;19:20. doi: 10.1186/s12943-020-1134-8 (PMC6993513; doi:10.1186/s12943-020-1134-8)
Supplement: Supplementary file 1 — Additional file 1: Table S1. The primer sequences used for real-time quantitative PCR. [file 12943_2020_1134_MOESM1_ESM.docx]

Supplementary Table S1

| name | Upper primer（5’-3’） | Down primer（5’-3’） |
| --- | --- | --- |
| GAPDH | GGACCTGACCTGCCGTCTAG | GTAGCCCAGGATGCCCTTGA |
| U6 | CTCGCTTCGGCAGCACA | AACGCTTCACGAATTTGCGT |
| circ_001680 | TCTACTTTGAGTGCTGTCTCCA | GTTAACATTATTATAACCCTGCTCAGA |
| miR-340 | AACTGTTTGCAGAGGAAACTGA | Universal primers |
| BMI1 | CCACCTGATGTGTGTGCTTTG | TTCAGTAGTGGTCTGGTCTTGT |
| CD44 | CTGCCGCTTTGCAGGTGTA | CATTGTGGGCAAGGTGCTATT |
| CD133 | AGTCGGAAACTGGCAGATAGC | GGTAGTGTTGTACTGGGCCAAT |
| Sox2 | GCCGAGTGGAAACTTTTGTCG | GGCAGCGTGTACTTATCCTTCT |
| Bio-circ_001680-probe | Bio-5’-CATGGACATGATCTTCTTTATAA-3′-Bio | |
| Bio-miR-340-probe | Bio-5’-TTAGTCAGAGTAACGAAATATT-3′-Bio | |
| Digo-circ_001680-probe | Digo-5’-CATGGACATGATCTTCTTTATAA-3′-Digo | |
| Bio-BMI1-probe | Bio-5’-ATTTGCTTTCTTTTGTAGTGACATTAAATT-3′-Bio | |
