# Supplementary figures and images for: Hsa_circ_001680 affects the proliferation and migration of CRC and mediates its chemoresistance by regulating BMI1 through miR-340
Source: Mol Cancer. 2020 Jan 31;19:20. doi: 10.1186/s12943-020-1134-8 (PMC6993513; doi:10.1186/s12943-020-1134-8)

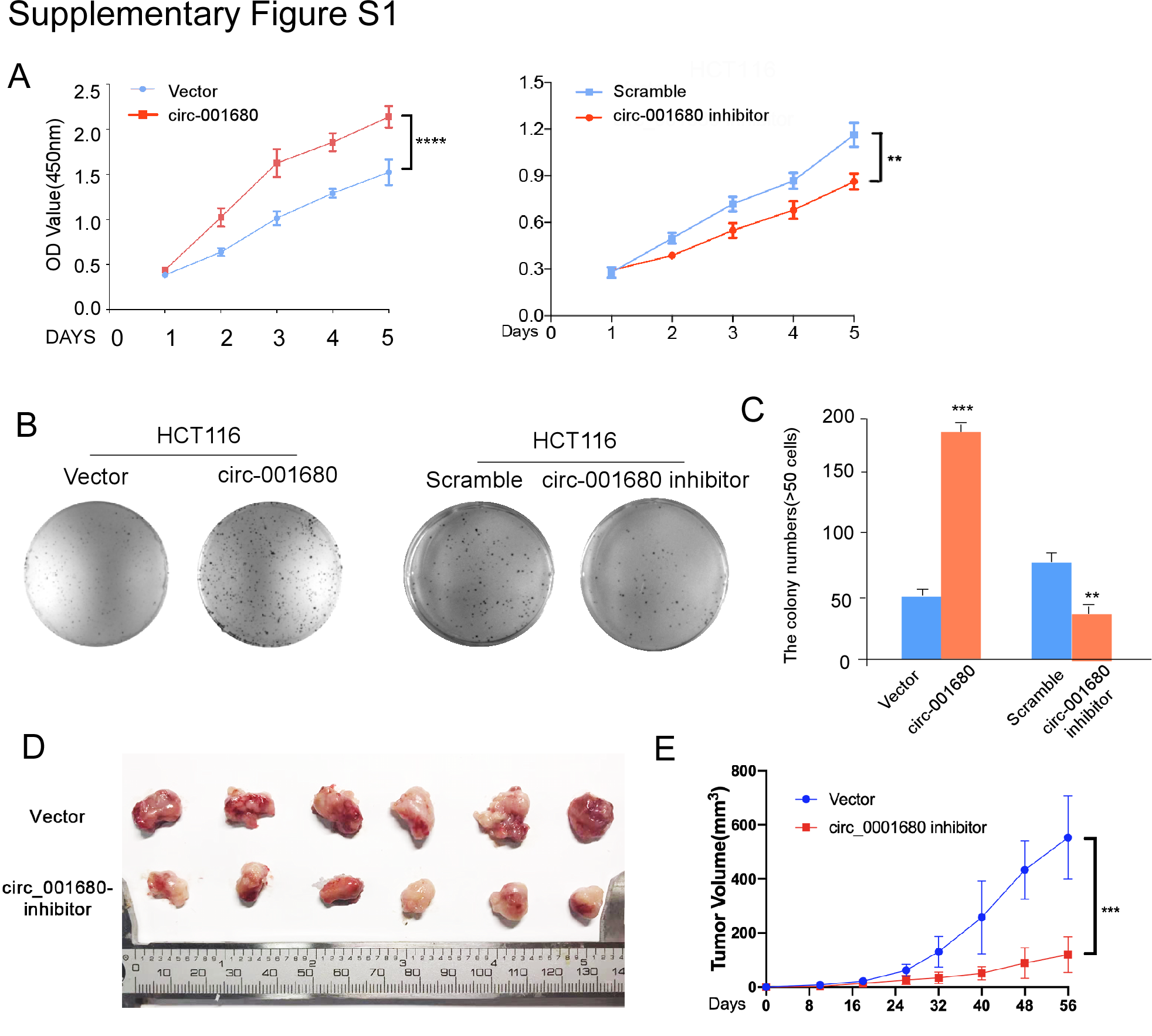

Supplement: Supplementary file 2 — Additional file 2: Figure S1. Circ_001680 affected the proliferation ability of CRC cells in vitro. (A) CCK8 assay results comparing the effects of circ_001680 on cell growth between the HCT116 vector and HCT116 circ_001680 groups (left) and between the HCT116 scramble and HCT116 circ_001680 inhibitor groups (right). (B) Representative images of colony formation in the indicated cells. The colonies containing > 50 cells were scored. (C) The number of colonies in an entire well was counted. The error bars represent the mean ± SD from three independent experiments (**p < 0.01, ***p < 0.005). (D) Image of the tumor xenograft model (n = 6). (E) Tumor growth curve. The error bars represent the means ± SD from three independent experiments (***p < 0.005). [file 12943_2020_1134_MOESM2_ESM.tif]

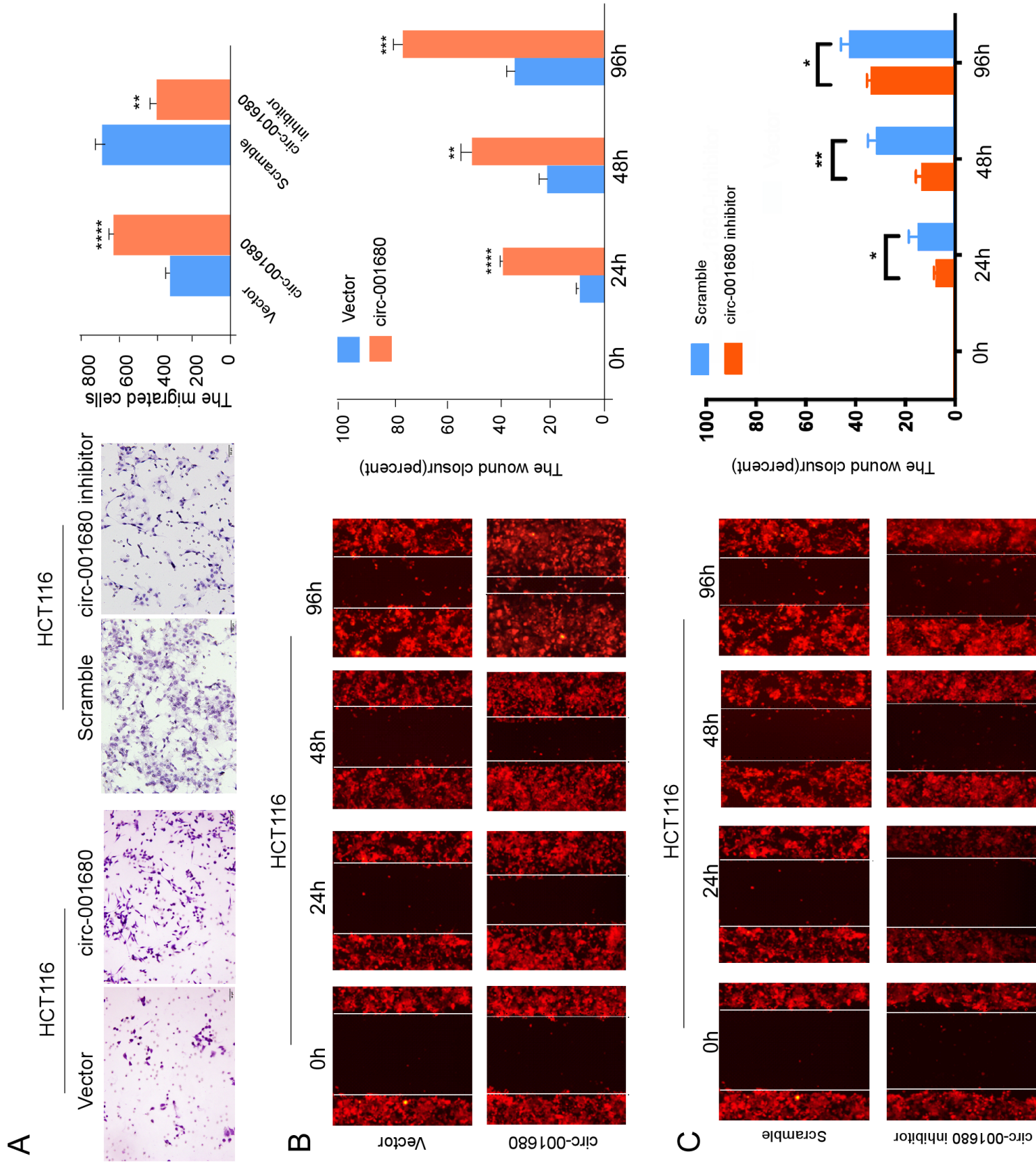

Supplement: Supplementary file 3 — Additional file 3: Figure S2. Circ_001680 affected the growth ability of CRC in vitro. (A) Representative transwell images of the effect of circ_001680 on the migration of the indicated cells (left). Statistical analysis of the transwell assay results (right). (B) The wound healing assay results showing divergent migration capacities at 4 regular intervals in the indicated cells (left); the statistical analysis is shown on the right. The error bars represent the means ± SDs from three independent experiments. *p < 0.05, **p < 0.01, ***p < 0.005. [file 12943_2020_1134_MOESM3_ESM.pdf]

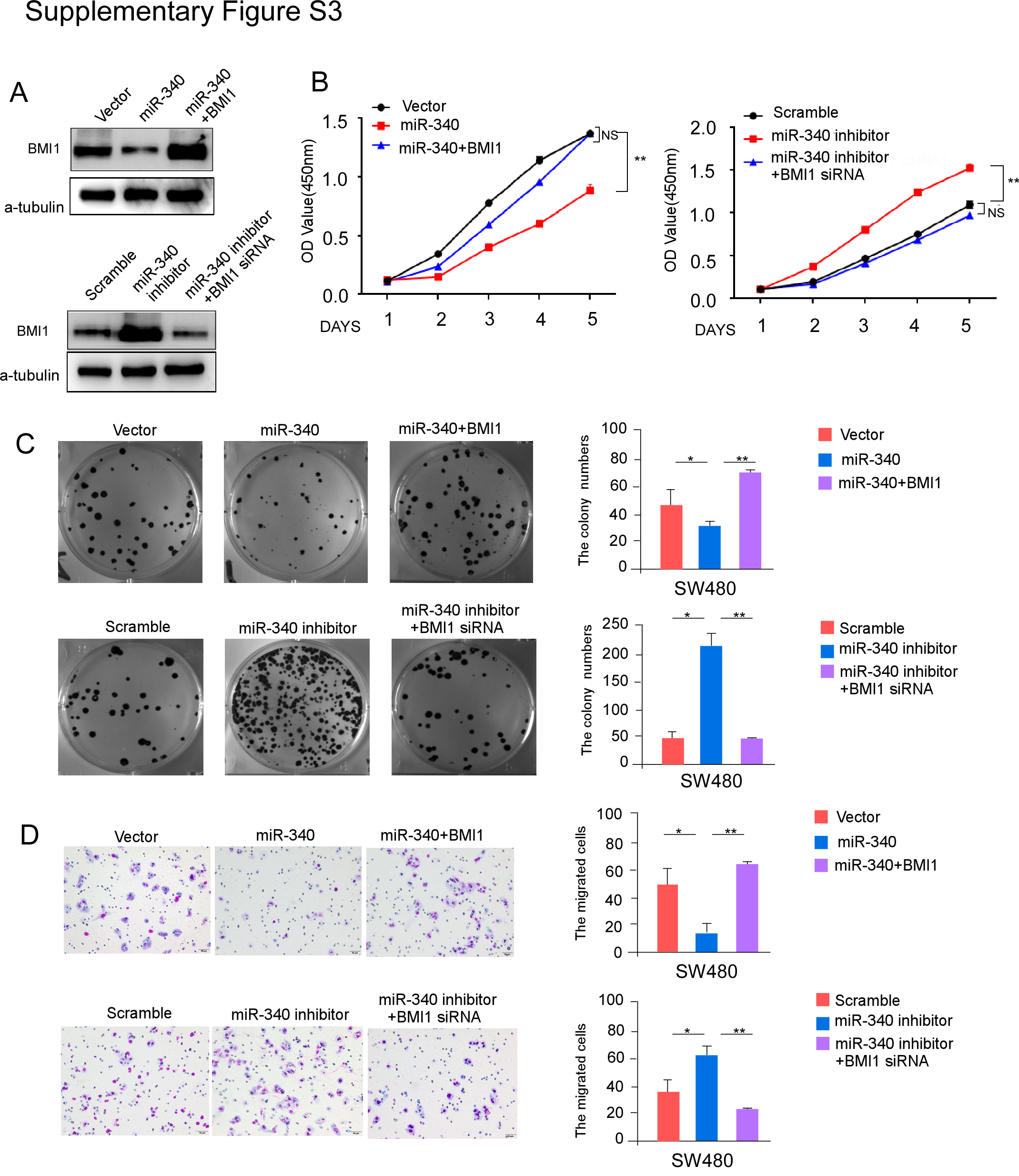

Supplement: Supplementary file 4 — Additional file 4: Figure S3. BMI1 is the bona fide effector of miR-340 in vivo. (A) Western blot analysis of BMI1 in the indicated cells. (B) Representative growth of the indicated cells as determined by the CCK8 assay. (C) Representative images (left) and statistical chart (right) of the colony formation assay in the indicated cells. (D) Representative images (left) and statistical chart (right) of migrated cells across the transwell chamber in indicated cells. The error bars represent the means ± SD from three independent experiments. *p < 0.05, **p < 0.01. [file 12943_2020_1134_MOESM4_ESM.tiff]

Supplementary Figure S4

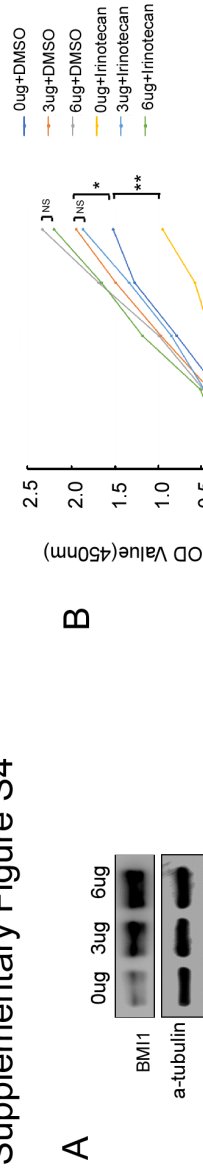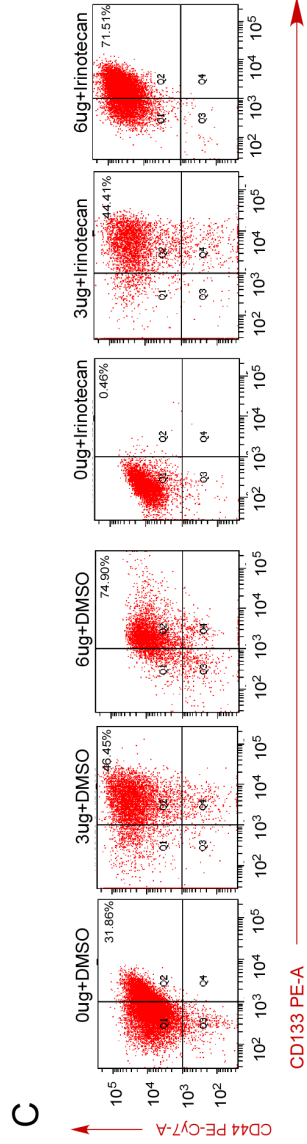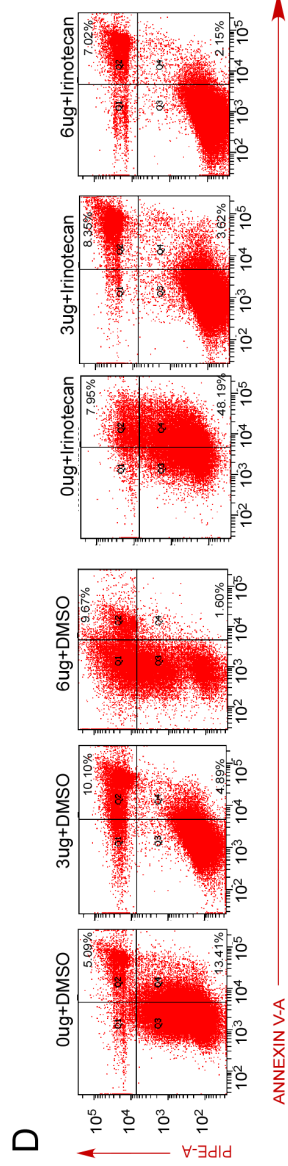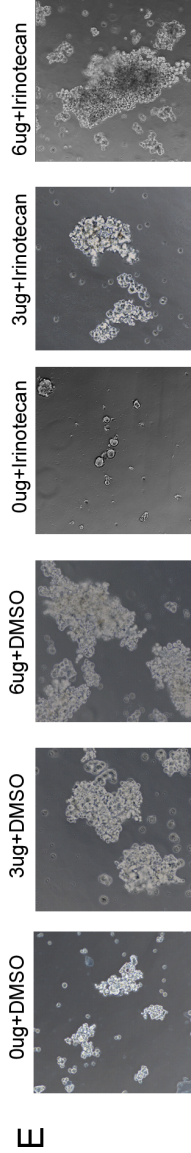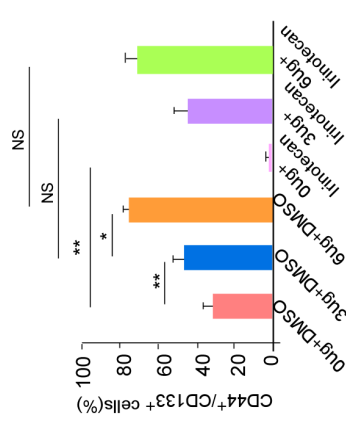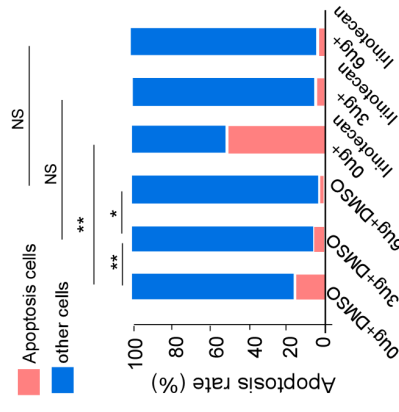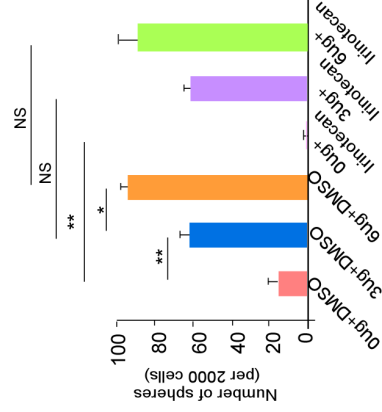

Supplement: Supplementary file 5 — Additional file 5: Figure S4. Irinotecan resistance induced by different concentration gradients of BMI1 in CRC cells. (A) SW480 cells were transiently transfected with the indicated amounts of BMI1. The protein level of BMI1 was detected by Western blotting after 48 h. (B) Representative growth of the indicated cells as determined by a CCK8 assay. (C) The number of subpopulation cells with the CD44+/CD133+ phenotype in the indicated SW480 cells (left). Quantification of cells with the CD44+/CD133+ phenotype is shown in the histogram (right). (D) Apoptosis assay of the indicated cells by flow cytometry (left). Statistical analysis of the flow cytometry results (right). (E) Typical images from the sphere formation assay of the indicated lentivirus-infected cells treated with or without irinotecan. The error bars represent the mean ± SD from three independent experiments. **p < 0.01, ***p < 0.005, ****p < 0.001. [file 12943_2020_1134_MOESM5_ESM.pdf]

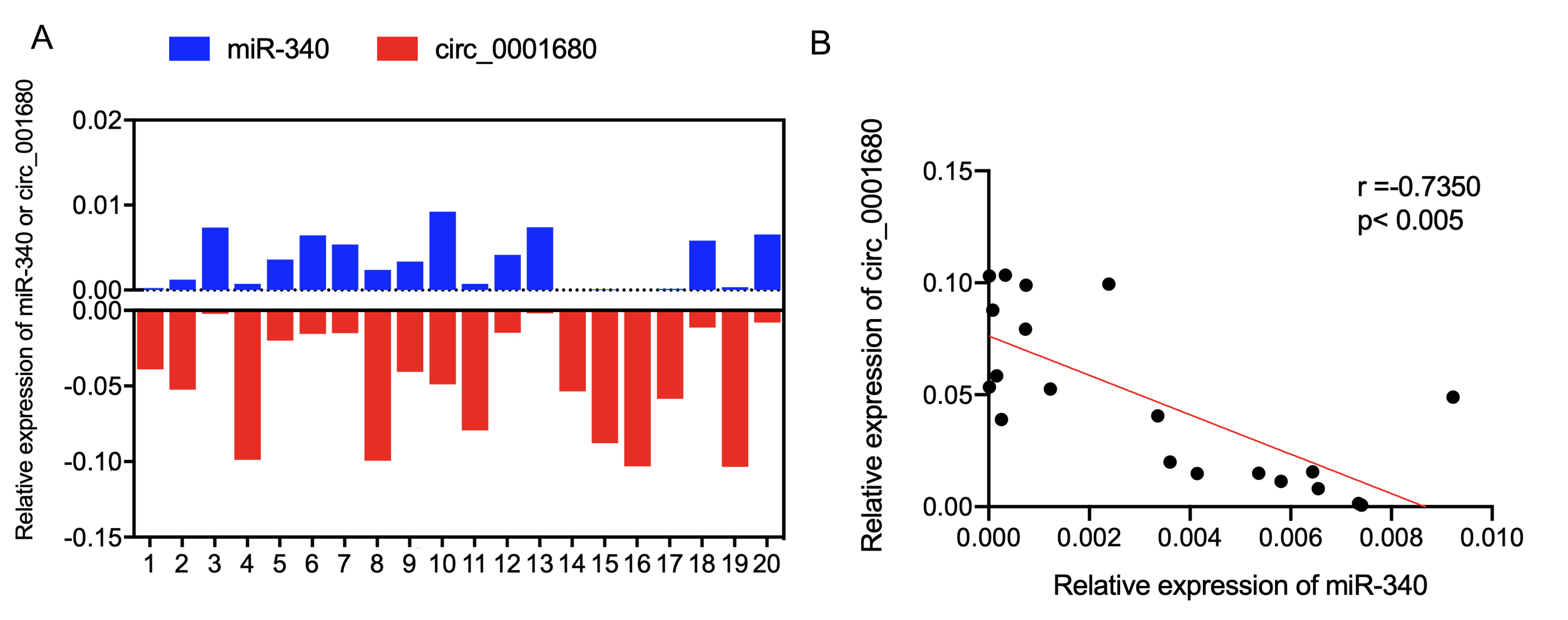

Supplement: Supplementary file 6 — Additional file 6: Figure S5. circ_001680 was negatively correlated with miR-340. (A)qRT-PCR analysis of circ_001680 and miR-340 expression in 20 fresh human colorectal cancer tissues. (B) Correlation analysis showed that the expression of miR-340 is negatively correlated with circ_001680. [file 12943_2020_1134_MOESM6_ESM.tif]
